# Very-low-dose aspirin and surveillance colonoscopy is cost-effective in secondary prevention of colorectal cancer in individuals with advanced adenomas: network meta-analysis and cost-effectiveness analysis

**DOI:** 10.1186/s12876-021-01715-7

**Published:** 2021-03-20

**Authors:** Sajesh K. Veettil, Siang Tong Kew, Kean Ghee Lim, Pochamana Phisalprapa, Suresh Kumar, Yeong Yeh Lee, Nathorn Chaiyakunapruk

**Affiliations:** 1 Department of Pharmacotherapy, College of Pharmacy, The University of Utah, 30 2000 E, Salt Lake City, UT 84112 USA; 2 Department of Pharmacy Practice, School of Pharmacy, International Medical University, Bukit Jalil, 57000 Kuala Lumpur, Malaysia; 3 Department of Internal Medicine, School of Medicine, International Medical University, International Medical University, Bukit Jalil, 57000 Kuala Lumpur, Malaysia; 4 Department of Surgery, International Medical University, Negeri Sembilan, Jalan Rasah, 70300 Seremban, Malaysia; 5 Division of Ambulatory Medicine, Department of Medicine, Faculty of Medicine Siriraj Hospital, Mahidol University, Bangkok, 10700 Thailand; 6 School of Medical Sciences, Universiti Sains Malaysia, Kota Bharu, Malaysia; 7 Gut Research Group, Faculty of Medicine, National University of Malaysia, Kuala Lumpur, Malaysia; 8 School of Pharmacy, Monash University Malaysia, Bandar Sunway, 47500 Subang Jaya, Selangor Malaysia

**Keywords:** Colorectal cancer, Colorectal adenomas, Chemoprevention, Aspirin, Surveillance colonoscopy, Network meta-analysis, Cost-effectiveness analysis

## Abstract

**Background:**

Individuals with advanced colorectal adenomas (ACAs) are at high risk for colorectal cancer (CRC), and it is unclear which chemopreventive agent (CPA) is safe and cost-effective for secondary prevention. We aimed to determine, firstly, the most suitable CPA using network meta-analysis (NMA) and secondly, cost-effectiveness of CPA with or without surveillance colonoscopy (SC).

**Methods:**

Systematic review and NMA of randomised controlled trials were performed, and the most suitable CPA was chosen based on efficacy and the most favourable risk–benefit profile. The economic benefits of CPA alone, 3 yearly SC alone, and a combination of CPA and SC were determined using the cost-effectiveness analysis (CEA) in the Malaysian health-care perspective. Outcomes were reported as incremental cost-effectiveness ratios (ICERs) in 2018 US Dollars ($) per quality-adjusted life-year (QALY), and life-years (LYs) gained.

**Results:**

According to NMA, the risk–benefit profile favours the use of aspirin at very-low-dose (ASAVLD, ≤ 100 mg/day) for secondary prevention in individuals with previous ACAs. Celecoxib is the most effective CPA but the cardiovascular adverse events are of concern. According to CEA, the combination strategy (ASAVLD with 3-yearly SC) was cost-saving and dominates its competitors as the best buy option. The probability of being cost-effective for ASAVLD alone, 3-yearly SC alone, and combination strategy were 22%, 26%, and 53%, respectively. Extending the SC interval to five years in combination strategy was more cost-effective when compared to 3-yearly SC alone (ICER of $484/LY gain and $1875/QALY). However, extending to ten years in combination strategy was not cost-effective.

**Conclusion:**

ASAVLD combined with 3-yearly SC in individuals with ACAs may be a cost-effective strategy for CRC prevention. An extension of SC intervals to five years can be considered in resource-limited countries.

**Supplementary Information:**

The online version contains supplementary material available at 10.1186/s12876-021-01715-7.

## Background

Colorectal cancer (CRC) is the third most commonly diagnosed malignant neoplasm and the second leading cause of cancer death worldwide [1]. Similarly, CRC is on the rise in Malaysia, and from the most recent data from the national cancer registry indicate that CRC is now the second most common cancer and the commonest among males [2]. Colorectal adenoma is a known premalignant condition, but individuals with high risk of CRC are those with advanced colorectal adenomas (ACAs), typically defined as 1 cm or larger, and/or have villous component and/or high-grade dysplasia [3].

In high-risk individuals, it is attractive to have chemopreventive agents (CPAs) which are effective in protecting them from getting a recurrence of ACAs after initial polypectomy [4, 5]. In addition, the CPA should be ideally free from adverse events but also cost-effective considering its long-term use. There are several candidates, including aspirin and celecoxib but it is unknown if these agents fullfill all the requirements described above [6, 7]. The US Preventive Services Task Force (USPSTF) guideline supports the use of very-low-dose aspirin (ASAVLD, ≤ 100 mg/day) for primary chemoprevention of CRC [8] but secondary chemoprevention in particular patients with a previous history of ACAs is unclear. That could be in part because of the absence of data informing the relative efficacy of aspirin at different doses (especially at a dose suggested by the USPSTF for the primary prevention [9]) on reducing the recurrence of ACAs. Previous systematic reviews and network meta-analyses (NMAs) also did not investigate this gap in the literature [4, 5]. Additional RCTs have since become available allowing re-examination of the existing evidence [10–12].

Meanwhile, screening colonoscopy with resection of detectable adenomas followed by interval surveillance colonoscopy (SC) is typically regarded as the ideal preventive approach [13]. Unfortunately, SC has several limitations including cost, suboptimal adherence, limited access, possible complications and the risk of missing adenomas [14]. Hence, increasing attention is being given to chemoprevention as a substitute for routine SC, or alternatively a combination strategy using both approaches. It is unknown if chemoprevention may allow extension of surveillance intervals from the recommended 3-yearly to 5-yearly or 10-yearly, and such an approach may be cost-effective in countries with limited health-care resources.

Therefore, our objectives were first to identify the ‘ideal’ CPA for secondary prevention using NMA techniques, second to investigate the cost-effectiveness of that CPA alone, SC alone or combination strategy for prevention of new CRCs in a high-risk population with a previous history of ACAs, and third to determine if an extension of surveillance intervals to 5-yearly and 10-yearly is feasible in terms of cost-effectiveness. For the last 2 objectives, a health economic model was developed using data from our NMA as well as population and health-care settings of Malaysia, a developing nation with an estimated population of 30 million in the South-east Asia region.

## Methods

### Systematic review and network meta-analysis

First, systematic review with network meta-analysis (NMA) was performed to identify a CPA with the most favourable risk–benefit profiles to be further examined in the proposed cost-effectiveness model. The NMA was registered with PROSPERO (CRD42015025849) [15] and reported according to the Preferred Reporting Items for Systematic Reviews and Meta-Analyses (PRISMA) extension statement [16]. The primary efficacy outcome was the incidence of recurrent ACAs. Safety outcomes were the incidence of serious adverse events (SAEs) and cardiovascular (CV) events. Definitions of efficacy and safety outcomes are provided in Additional file 1: 1.1. Search strategy and study selection are described in Additional file 1: 1.2. Studies included were RCTs with a duration of treatment of at least one year. The intervention was any CPAs including aspirin (high-dose or ASAHD > 325 mg/day, low-dose or ASALD 100–325 mg/day and very-low-dose or ASAVLD ≤ 100 mg/day) [9], celecoxib, calcium and vitamin D, alone or in combination at different doses. Comparator intervention was another CPA or placebo. Inclusion criteria are provided in Additional file 1: 1.3. Data extraction and quality assessment are provided in Additional file 1: 1.4.

Details of statistical analysis are provided in Additional file 1: 1.5. The outcome measure was described using risk ratio (RR) and 95% confidence interval (CI). For direct comparisons between interventions, a standard pairwise meta-analysis was performed by using the random-effects model [17]. Trial sequential analyses (TSAs) were performed to assess the risk of random errors in pairwise meta-analyses [18] (Additional file 1: 1.5.1). Random effects NMA using consistency model was applied in comparison of all interventions using direct and indirect estimates [19] Inconsistency assumption was evaluated using a global inconsistency test by fitting design-by-treatment in the inconsistency model [20]. In order to rank intervention hierarchy, surface under the cumulative ranking (SUCRA) curves were derived [21] Publication bias was examined with comparison-adjusted funnel plot [22] To assess the robustness of primary outcome, multiple sensitivity analyses were performed (Additional file 1: 1.5). For statistical analysis and graph generation, Stata version 15.1 (StataCorp, College Station, TX, USA) was utilised. The risk–benefit integrated analysis was used to review the potential benefits and risks of CPAs (Additional file 1: 1.5.2). The quality of evidence from NMA was evaluated using the Grading of Recommendations, Assessment, Development and Evaluation (GRADE) approach [23].

### Development of cost-effectiveness analysis (CEA) model

Based on evidence generated from the NMA, a Markov model (detailed description of the model and its assumptions are provided in Additional file 1: 2.1) was then developed to evaluate the long-term clinical and economic benefits of using the chosen CPAs. The NMA choice of CPAs and population characteristics in this analysis are described in Additional file 1: 2.2.

A hypothetical cohort of 100,000 individuals aged 50 years and above began the simulation in an adenoma-cancer-free state (that is, normal colon), with an assumption that the baseline colonoscopy was 100% successful in removing all adenomas after polypectomy. The model simulated a series of sequential transitions of 12 health states based on yearly probabilities i.e. 1) normal colon, 2) low-risk state, 3) high-risk state, 4) to 7) CRC stage I-IV (pre-clinical), 8) to 11) CRC stage I-IV (clinical), and 12) death (definitions of these health states are provided in Additional file 1: 2.3). The study cohort was subjected to the following four CRC prevention strategies: 1) no intervention, 2) SC every 3 years after baseline colonoscopy, 3) the NMA-chosen CPA alone; and 4) combination strategy (NMA-chosen CPA with 3-yearly SC as described above). Outcomes were assessed in the provider perspective and reported as incremental cost-effectiveness ratios (ICERs) in 2018 United States Dollar ($) per quality-adjusted life year (QALY) gained and life-years (LYs) gained. Costs, outcomes, and utilities were discounted at a rate of 3% from the point at which the individuals have begun interventions. The time horizon of the model was lifetime with a cycle length of 1 year. The costs and outcomes accrued beyond the age of 50 years were estimated, and the simulation continued until the remaining lifetime of the study cohort. The Malaysian ceiling threshold of societal willingness to pay (WTP; cost-effectiveness threshold) of $7024/QALY was used to interpret the cost-effectiveness analysis (CEA) [24].

### Input parameters in the CEA model

A summary of all input parameters used in the CEA model is provided in Table 1. The annual probability of recurrence of ‘low-risk’ adenomas after index polypectomy was derived from the pooled estimates of the National Cancer Institute (NCI) pooling project [25]. The annual probability of recurrence of ‘high-risk’ adenomas was calculated based on a meta-analysis using four sets of data reported by the NCI pooling project [25]. The probabilities of developing pre-clinical early-stage CRC (stage-I) were assumed to be age-dependent [26]; these probabilities were obtained from annual transition rates reported in birth cohort analyses from the German nationwide screening colonoscopy registry [26, 27]. The transitional probabilities of all other pre-clinical stages of CRC were as previously reported (estimated by calibration of the NCI data from 1973 through 1999 on cancer incidence and stage distribution [28]). Frazier et al. [29] Probabilities of subjects who were initially at pre-clinical stages and subsequently detected in clinical stages would depend if symptoms developed and also diagnostic accuracy of SC or other tests [29].

**Table 1:** Summary of input parameters

Parameter	Base case	SE or range	Distribution	Source/references
*Annual transition probabilities*				
Normal to low-risk	0.1976	0.0044	Beta	Based on the National Cancer Institute pooling project [25] (S 3.1)
Low-risk state to high-risk	0.0890	0.0028	Beta	Meta-analysis of 4 data sets of population with high-risk adenomas at baseline (S 3.2)
High-risk to CRC1pre	ASR	NA	NA	Birth cohort analyses from German screening colonoscopy registry [26, 27] (S 3.3)
CRC1pre to CRC2pre	0.2800	0.0357	Beta	Estimated by calibration to the National Cancer Institute data statistics [28], 1973–1999
CRC2pre to CRC3pre	0.2800	0.0357	Beta	
CRC3pre to CRC pre	0.6300	0.1405	Beta	
CRC1pre to CRC1cli (by symptoms)	0.0700	0.0300	Beta	Reported in an economic evaluation by Frazier AL et al. [29]
CRC2pre to CRC2cli (by symptoms)	0.2500	0.0577	Beta	
CRC3 pre to CRC3cli (by symptoms)	0.5500	0.0577	Beta	
CRC4pre to CRC4cli (by symptoms)	0.8500	0.0763	Beta	
CRC1cli to dead	0.0575	0.0087	Beta	Based on meta-analyses of five studies reported survival data of CRC at different stages in Malaysia (S 3.8)
CRC2cli to dead	0.0684	0.0099	Beta	
CRC3cli to dead	0.0973	0.0132	Beta	
CRC4cli to dead	0.1589	0.0666	Beta	
*Effectiveness: every 3-year colonoscopy*				
Low-risk state to normal	0.5800	0.0178	Beta	Based on meta-analyses of per-patient miss rate (S 3.4.1–2)
High-risk state to normal or low-risk state	0.9200	0.0204	Beta	
CRC1pre to CRC1cli	0.9470	0.013	Beta	Available from a meta-analysis by Pickhardt PJ et al. [30]
CRC2pre to CRC2cli	0.9470	0.013	Beta	
CRC3pre to CRC3cli	0.9800	0.9500–0.9900	Uniform	Available from an economic evaluation from the National Institute for Health Research (NIHR) (S 3.5)
CRC4pre to CRC4cli	0.9800	0.9600–1.0000	Uniform	
*Relative risk (RR) of benefits associated with ASAVLD*				
Normal to low-risk	0.86	0.0740	Normal	Meta-analyses of two aspirin chemoprevention RCTs [39, 40] (S 3.6)
Low-risk to high-risk	0.59	0.1352	Normal	
RR of CV mortality	0.92	0.0536	Normal	Reported in a recent network meta-analysis by Veettil et al. [37]
*Harms associated with interventions*				
Intolerability due to initial side effects of ASAVLD	0.052	0.025–0.200	Uniform	Derived from an aspirin chemoprevention trial [39]
Major bleeding (any) due to ASAVLD per year	0.0022	0.0005	Beta	Available from a meta-analysis of nine primary CV disease prevention trials [34] (S 3.7.1)
Major GI bleeding due to ASAVLD	0.0011	0.0003	Beta	Available from the systematic review undertaken for the USPSTF [35, 36] and Veettil et al. [37] (S 3.7.2)
Ulcer due to ASAVLD	0.0018	0.0002	Beta	
Dyspepsia due to ASAVLD	0.1880	0.0800	Beta	
Perforation due to colonoscopy (with or without polypectomy)	0.0004	0.00008	Beta	Based on a systematic review undertaken for the USPSTF by Lin JS et al. [31]
Major bleeding due to colonoscopy	0.0008	0.0002	Beta	
Mortality due to perforation	0.0582	0.0100	Beta	Available from a large population-based cohort study by Gatto NM et al. [38]
Mortality due to major bleeding events	0.0600	0.0100–0.1600	Uniform	Reported in a recent network meta-analysis by Veettil et al. [37]
*Utility values*				
Non-CRC states	0.8300	0.0500	Beta	Based on a population based cross-sectional study using EQ-5D instrument [40] (S 3.9)
CRC I	0.7400	0.0260	Beta	Ness et al. [39]
CRC II	0.7400	0.0260	Beta	
CRC III	0.6700	0.0289	Beta	
CRC IV	0.2500	0.0551	Beta	
Colonoscopy (disutility)	0.0025	NA	NA	Reported in an economic evaluation by Saini SD et al. [57] (S 3.9)
Major GI bleeding/peptic ulcer due to ASAVLD (1 month)	0.46	NA	NA	Based on the analysis undertaken for the NICE osteoarthritis guidelines (S 3.9)
Dyspepsia (1 month)	0.73	NA	NA	
*Base case assumptions*				
Annual discount rate for costs and outcomes	0.03	NA	NA	Pharmacoeconomic guideline, Malaysia (https://www.pharmacy.gov.my/v2/en/documents/pharmacoeconomic-guideline-malaysia.html)
Compliance to surveillance colonoscopy	60%	30–100%	NA	Taylor et al. [58]

ASAVLD, aspirin very-low-dose; ASR, age-specific rate; cli, clinical; CRC, colorectal cancer; NA, not applicable; pre, pre-clinical; SE, standard error

The sensitivity of diagnostic SC was obtained from a recent systematic review and meta-analysis of 25 studies [30]. The impact of SC on low- and high-risk adenomas was derived from per-patient miss rates (Additional file 1: 3.4) which were reasonably estimated based on meta-analyses of data from the Asian population (Additional file 1: Figs. 3.4.1-2). The probability of perforation (with or without polypectomy) and major bleeding due to colonoscopy was based upon the report of a systematic review undertaken for the USPSTF [31].

The RRs of developing low- or high-risk adenomas or adverse effects related to the use of CPAs were derived from RCTs [32, 33] and meta-analyses [34–37] (Additional file 1: 3.6–7). The annual probability of mortality from any causes was estimated using repository data from the Global Health Observatory data (Additional file 1: Table 3.8.1). The probabilities of deaths for each stage of CRCs were calculated based on a meta-analysis of 5 studies in Malaysia (Additional file 1: Table 3.8.2). Probability of death following perforation was obtained from a large population-based cohort study [38]. Probability of death following major bleeding events, and the RRs of CV mortality on ASAVLD was as previously reported in a systematic review [37].

The respective stage-specific utility scores for different stages of CRC were obtained from a study eliciting preferences for a hypothetical stage from individuals who had previously undergone polypectomy [39]. The utility of patients without CRC was obtained from a cross-sectional study of Malaysian adult population-based values for EQ-5D health states [40]. The impact on the quality of life from harms associated with colonoscopy and or use of the chosen CPA was also incorporated into the CEA model (Additional file 1: 3.9).

### Cost data in the CEA model

Cost estimates used within the CEA analysis have been derived from the amended Malaysia medical fee schedule 2013 [41], the consumer price guide database, data from the Ministry of Health of Malaysia [42], and other relevant literatures. Based on a standard operating procedure available from the Malaysia national guidelines, the total provider cost for initial treatment of CRC per year was obtained from a cost analysis conducted at a tertiary hospital in the country [43]. The lifetime costs of CRC were reasonably estimated based on the follow-up policies advocated by the Malaysian clinical practice guideline on CRC (Additional file 1: Table 3.10.1). A detailed description of all cost estimates used within the analysis is provided in Additional file 1: 3.10.2. All costs were converted using the consumer price index (CPI) (https://www.dosm.gov.my/v1/) and reported in 2018 US Dollars ($).

### Sensitivity analyses

One-way sensitivity analyses were performed to study the effects of altering parameters on the CEA findings. The 95% CI ranges were used whenever such data were available; but if absent, the ± 15% range was applied. Results were shown using the tornado diagrams to identify parameters with the most significant impact on the model results. A probabilistic sensitivity analysis (PSA) was also conducted to simultaneously examine the effects of all parameter uncertainties using a Monte Carlo simulation performed using Microsoft Excel 2003 (Microsoft Corp, Redmond, WA) [44] Results of the PSA were presented as cost-effectiveness acceptability curve.

## Results

### The Choice of CPA based on NMA

**Fig. 1:**
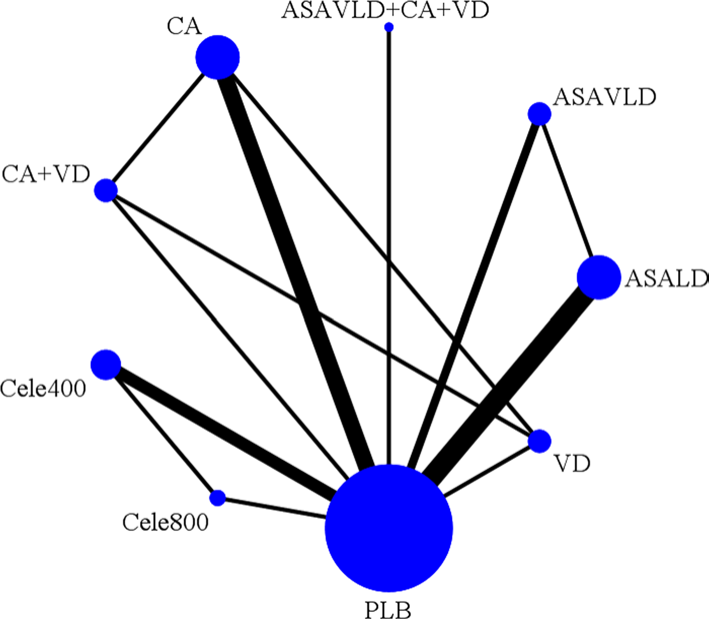
Network plot of chemopreventive agents tested in RCTs for recurrence of advanced colorectal adenomas. Connecting lines represent head-to-head (pairwise) comparisons, indicated by the connected nodes (size proportional to the number of studies). Line thickness is proportional to the number of studies comparing the two strategies. Abbreviations: ASALD, low-dose-aspirin; ASAVLD, very-low-dose-aspirin; Ca, calcium; Cele, celecoxib (400 mg and 800 mg daily), PLB, placebo; VD, vitamin D

**Fig. 2:**
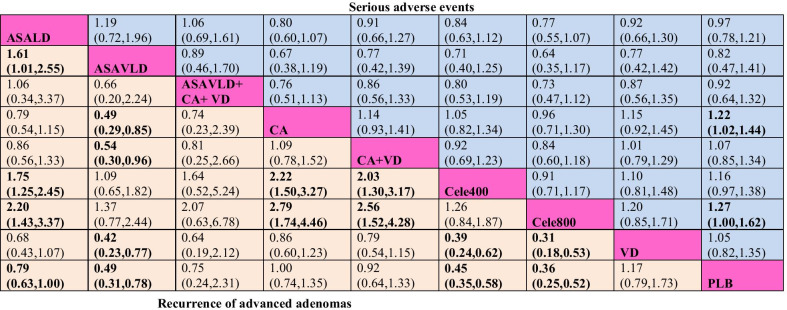
Comparative efficacy and safety of chemopreventive agents for recurrence of advanced adenomas in network meta-analysis. Note: Outcomes are expressed as risk ratio (RR, with 95% confidence interval). Comparisons should be read from left to right. RR < 1 indicates that the treatment specified in the column is more efficacious in preventing recurrent advanced adenomas. For risk of serious adverse events, RR < 1 indicates that the treatment specified in the row is safer. Bold cells are significant. Abbreviations: ASALD, low-dose-aspirin; ASAVLD, very-low-dose-aspirin; Ca, calcium; Cele, celecoxib (400 mg and 800 mg daily), PLB, placebo; VD, vitamin D

A flow diagram depicting the search and selection process is provided in Additional file 1: 1.2.1. From a total of 4673 citations from the search strategy, 14 RCTs [10–12, 32, 33, 45–53] comparing nine interventions (including placebo) were evaluated in the NMA. Figure 1 shows the available direct comparisons and network of RCTs for the efficacy outcome. Additional file 1: Tables 4.1.1-2 describe the characteristics of all included RCTs. A summary of risk of bias assessment is presented in Additional file 1: 4.2. Treatment effects estimated from pairwise meta-analyses are presented in Additional file 1: 4.3. Treatment effects estimated from NMA for efficacy and safety outcomes are presented in Fig. 2. Detailed descriptions of the results of NMA for efficacy and safety outcomes are provided in Additional file 1:s 4.4-5, respectively.

Based on NMA, celecoxib 800 mg/day (RR, 0.36 [95% CI 0.25 to 0.52]) and celecoxib 400 mg/day (RR, 0.45 [95% CI 0.35 to 0.58]) were ranked best for preventing recurrence of ACAs compared to placebo, followed by ASAVLD (RR, 0.49 [95% CI 0.31 to 0.78]) and ASALD (RR, 0.79 [95% CI 0.63 to 1.00]). Based on comparative efficacy, none of these CPAs was superior over the others, except for ASALD (Fig. 2). Overall, the results were robust with respect to sensitivity analyses (Additional file 1: Table 4.4.2) and reasonably comparable to pairwise meta-analyses (Additional file 1: Fig. 4.4.2). TSAs based on pairwise meta-analyses (Additional file 1: 4.6) have demonstrated significant conclusive effects of ASAVLD and celecoxib but inconclusive for ASALD.

In the context of safety, with NMA, celecoxib 400 mg/day (RR, 1.16 [95% CI 0.97–1.38]), celecoxib 800 mg/day (RR, 1.27 [95% CI 1.00–1.60]), and calcium (RR, 1.22 [95% CI 1.02–1.44]) were associated with increased risk of SAEs. In addition, celecoxib (400–800 mg/day) was associated with increased risk of CV events (Additional file 1: 4.5). Based on NMA, ASAVLD was ranked safest among the CPAs.

No substantial inconsistency and small-study effects were identified in NMA (Additional file 1: 4.7). Overall based on GRADE, the quality of evidence for the primary outcome was rated as moderate for ASAVLD, low for ASALD but high for celecoxib (Additional file 1: 4.8).

From risk–benefit integrated analysis (Additional file 1: 4.9), the use of ASAVLD, compared to placebo could reduce 77 (95% CI 24–109) and 35 (95% CI 11–51) fewer ACA cases per 1000 patients with advanced and non-advanced adenomas at the baseline, respectively but also six fewer serious adverse events (SAEs). For celecoxib, despite yielding higher efficacy compared to ASAVLD, the overall risk of SAEs was unacceptable (Additional file 1: 4.9). ASAVLD had the most favourable risk–benefit profile and was the preferred CPA over celecoxib. A scatter plot based on SUCRA ranking for efficacy and safety is also provided in Additional file 1: 4.10.

### Cost-effectiveness of ASAVLD, 3-yearly SC or combination strategy

With the choice of CPA now confirmed after NMA, a CEA model was then developed to assess the cost-effectiveness of using ASAVLD alone, 3-yearly SC alone, and the combination strategy of ASAVLD and 3-yearly SC. The base-case analysis of this model has demonstrated that, when compared to no screening, ASAVLD alone, 3-yearly SC, and combination strategy were less costly and more effective (in the order of decreasing costs and increasing effectiveness) (Table 2). Among all strategies, the combination strategy was the most cost-saving and the best buy option. For a base-case assumption of 60% compliance to colonoscopy, our model predicted a reduction in new cases of CRC by 14%, 23%, and 72% for ASAVLD, 3-yearly SC, and combination strategy, respectively, compared to no surveillance (Additional file 1: 5.1).

**Table 2:** Base case results

Strategies	Total costs (USD)	LYs	QALYs	Incremental costs (USD)	Incremental LYs	Incremental QALYs	ICER (USD/LY gained)	ICER (USD/QALY gained)
No screening	5757	17.23	10.44	-	-	-	-	-
ASAVLD	5472	17.69	10.60	− 285	0.47	0.17	Dominated^a^	Dominated^a^
Colonoscopy	4296	18.03	10.81	− 1176	0.34	0.21	Dominated^a^	Dominated^a^
Colonoscopy and ASAVLD	3679	18.43	10.93	− 617	0.40	0.12	Cost-saving	Cost-saving

ASAVLD, very low dose aspirin; ICER, incremental cost-effectiveness ratio; LYs, life-years; QALYs, quality-adjusted life years; USD, US dollar

^a^Dominated by combination of colonoscopy and ASAVLD

**Fig. 3:**
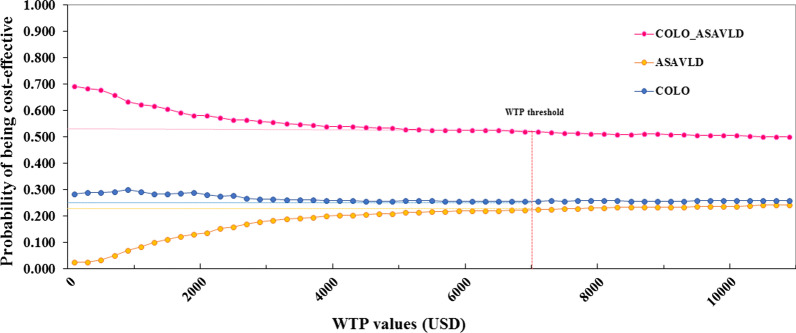
Cost-effectiveness acceptability curves of colorectal cancer preventive strategies. Abbreviations: ASAVLD: aspirin very-low-dose; COLO: colonoscopy; COLO_ASAVLD, combination strategy; USD, US dollar; WTP, willingness to pay

Tornado diagrams illustrating the one-way sensitivity analysis results of the combination strategy compared to no screening are presented in Additional file 1: Figs. 5.1-2. All parameters had no impact on cost-saving for the combination strategy, except for utility in non-CRC states. The results of PSA based on 1000 Monte Carlo simulations are illustrated using the cost-effectiveness plane (Additional file 1: Fig. 5.3) and acceptability curves (Fig. 3). The cost-effectiveness acceptability curves showed the superiority of the combination strategy over others for all the WTP values. Probabilities of being cost-effective for ASAVLD, 3-yearly-SC, and combination strategy were 22%, 26%, and 53%, respectively, based on the Malaysian ceiling threshold of social WTP of $7024 per QALY gained.

### Scenario analyses: extension of surveillance intervals in combination strategy

Effects on ICER from an extension of SC intervals in combination strategy to 5-yearly and 10-yearly are presented in Table 3. With 5-yearly SC, the combination strategy was associated with ICER of $484/LY gain and $1875/QALY gain. This was considered cost-effective based on the Malaysian ceiling threshold of social WTP. With 10-yearly SC, the combination strategy became less cost-effective with respect to LYs saved and QALYs gained. A similar trend was seen with respect to new cases of CRC that could be prevented in the simulated cohort over a lifetime. The model of combination strategy with 5-yearly SC predicted a reduction in CRC incidence by 55% (vs. no screening) and 42% (vs. 3-yearly SC) (Additional file 1: Table 5.2).

**Table 3:** Scenario analyses: Effect of extending colonoscopy surveillance intervals in the combination strategy

Strategies	Total costs (USD)	LYs	QALYs	Incremental costs (USD)	Incremental LYs	Incremental QALYs	ICER (USD/LY gained)	ICER (USD/QALY gained)
*Aspirin combined with colonoscopy at up to 5-year intervals*								
No screening	5757	17.23	10.44	-	-	-	-	-
ASAVLD	5472	17.69	10.60	− 285	0.47	0.17	Dominated^a^	Dominated^a^
Surveillance colonoscopy (3 years)	4296	18.03	10.81	− 1176	0.34	0.21	Cost-saving	Cost-saving
ASAVLD + surveillance colonoscopy (5 years)	4446	18.34	10.89	150	0.31	0.08	484 (Cost-effective)	1875 (Cost-effective)
*Aspirin combined with colonoscopy at up to 10-year intervals*								
No screening	5757	17.23	10.44	–	–	–	–	–
ASAVLD	5472	17.69	10.60	− 285	0.47	0.17	Dominated^a^	Dominated^a^
Surveillance colonoscopy (3 years)	4296	18.03	10.81	− 1176	0.34	0.21	Cost-saving	Cost-saving
ASAVLD + surveillance colonoscopy (10 years)	5878	18.11	10.78	1582	0.08	− 0.03	19,775 (Not cost-effective)	Dominated^a^
*Aspirin combined with colonoscopy every 5-year versus every 3-year*								
ASAVLD + surveillance colonoscopy (5 years)	4446	18.34	10.89	–	–	–	–	–
ASAVLD + surveillance colonoscopy (3 years)	3679	18.43	10.93	− 767	0.09	0.04	Cost-saving	Cost-saving

The Malaysian ceiling threshold of social willingness to pay (WTP) for interpretation of cost-effectiveness findings considered for the analysis was $7024 /QALY

ASAVLD, very low dose aspirin; ICER, incremental cost-effectiveness ratio; LYs, life-years; QALYs, quality-adjusted life years; USD, US dollar

^a^Dominated by surveillance colonoscopy (3-year)

## Discussion

Based on systematic review and NMA, we are able to conclude that ASAVLD is probably the safest although not the most effective CPA for prevention of recurrence of ACAs among individuals with a previous history of colorectal adenomas. Celecoxib is the most effective CPA, but the CV adverse events are of great concern. Moreover, the protective effect of celecoxib does not persist after its withdrawal [7]. The risk–benefit profile favours the use of ASAVLD, especially for those with a history of ACAs. Therefore, taken together with the risk–benefit analysis, there is moderate-quality evidence to support the choice of ASAVLD and in our subsequent CEA but also its long-term clinical benefit in combination with SC.

From the CEA, combination strategy (ASAVLD with 3-yearly SC) was the more cost-effective and the best-buy option with significant gains in LYs and QALYs. Moreover, a 63% reduction in the occurrence of new CRC cases was observed with the combination strategy compared to 3-yearly SC alone. Furthermore, ASAVLD has a positive impact on the prevention of cardiovascular events, and this is added benefit besides CRC reduction at fewer adverse outcomes. For countries with limited health-care resources including Malaysia, extending surveillance intervals to 5-yearly or 10-yearly may reduce health costs, however, in our scenario analysis, this was the case. For 5-yearly SC, while costlier, it could be the more cost-effective strategy in the Malaysian setting. However, we found 10-yearly SC was not cost-effective. Our findings may be applicable to countries with similar WTP thresholds as Malaysia [54].

Our findings are unique as none of the previous studies specifically evaluated the effectiveness of aspirin at very-low-dose in high-risk individuals with a history of advanced adenomas. Other differences include the following: 1) previous analyses [55, 56] have involved individuals with a history of ‘any’ colorectal adenomas, i.e. including non-advanced adenomas with a lesser risk for CRC and hence, we observed a higher number of new CRC cases in our model compared to others (e.g., 6.4% vs. 5.5% [55]) 2) the duration of surveillance colonoscopy was up to 75 years [13] in our model rather than lifetime [55, 57] or intermittent [52] in others, and 3) our model opted for 60% compliance with SC, [58] as opposed to 80–100% in previous chemoprevention models [55, 56], which, in our opinion, is not realistic in the real-world practice. The National Polyp Study suggested that SC should prevent at least 75% of all CRCs [59], and in our model, we predicted a reduction of 23%–57% for new or early-stage CRCs and 81% for late-stage CRCs.

When formulating the aspirin chemoprevention policy with SC, it is important to take into account the way in which aspirin chemoprevention may be implemented. Aspirin chemoprevention is more feasible in terms of human resources and budgetary burdens. Weighing the benefits of ASAVLD against the potential harms is of particular relevance in the chemoprevention setting. The use of aspirin needs shared decision making with patients but also comprehensive understanding of patients’ values and preferences. However, there are other factors relevant to the implementation of this strategy, including the budget impact, feasibility, and ethical and social implications that need to be considered for decision making. At present time, our findings are likely more applicable for countries with established colonoscopy screening programs and regular post-polypectomy surveillance. However, individuals from countries without colonoscopy screening programs but have been identified at increased risk due to advanced adenomas during any endoscopic examinations can still apply these findings, although the number of such individuals is expected to be minimal. Over the last ten years, an increase in screening rates in the general population has been observed in Malaysia [2]. This could further increase the burden of surveillance colonoscopy over time due to the scarcity of resources. Hence, the findings from this analysis have potential applications in the Malaysian setting and other lower-middle-income countries where resources are limited for the surveillance programs.

There are some limitations. First, CEA did not include the indirect costs of CRCs due to a lack of published data. Second, there is evidence for suboptimal efficacy of screening colonoscopy for proximal CRCs [60–62], which is currently the principal target of aspirin chemoprevention, but we did not consider the location of CRCs in our model. Third, the annual probabilities of input parameters (especially utility parameters, benefits, and harms associated with ASAVLD) were assumed to be constant over time but more likely is that with increasing age there would be a decrease in utility values and increase in risks of aspirin and colonoscopy-related morbidities. Fourth, the impact of ASAVLD on other cancers was not explored, and this could be a topic for future research. Fifth, for our base‐case analysis, we assumed that the initial colonoscopy was 100% successful in removing all adenomas. Unfortunately, this is not the case in real life, especially for the right colon. Lastly, our results are best replicated in future using the RCT and prospective designs, however, such studies are likely expensive and take a long time to complete.

## Conclusions

In conclusion, ASAVLD in combination with 3-yearly SC may be considered a cost-effective and safe strategy to prevent CRCs among high-risk individuals with a previous history of ACAs. For individuals already receiving ASAVLD, extension from 3-yearly to 5-yearly SC could be considered in the setting of limited resources.

## Supplementary Information

**Additional file 1**. Supporting information.

## Data Availability

All data generated or analyzed during this study was taken from published RCTs, systematic reviews, and other relavent literatures and are included in this article (and its supplementary information files).

## Abbreviations

ACAs: Advanced colorectal adenomas
ASALD: Aspirin at low-dose
ASAVLD: Aspirin at very-low-dose (≤ 100 mg/day)
CEA: Cost-effectiveness analysis
CPA: Chemopreventive agent
CPI: Consumer price index
CRC: Colorectal cancer
CV: Cardiovascular
GRADE: The Grading of Recommendations, Assessment, Development and Evaluation
ICER: Incremental cost-effectiveness ratio
LY: Life-years
LYG: Life-years gained
NCI: National Cancer Institute
NMA: Network meta-analysis
NSAIDs: Nonsteroidal anti-inflammatory drugs
PRISMA: Preferred reporting items for systematic reviews and meta-analyses
PSA: Probabilistic sensitivity analysis
QALY: Quality-adjusted life-year
RCT: Randomised controlled trial
RR: Risk ratio
SAE: Serious adverse event
SC: Surveillance colonoscopy
SUCRA: Surface under the cumulative ranking
TSA: Trial sequential analysis
US: United States
USPSTF: US Preventive Services Task Force
WTP: Willingness to pay threshold/ cost-effectiveness threshold

## Declarations

### Guarantor of the article

Professor Nathorn Chaiyakunapruk.
