# Supplementary material for: Very-low-dose aspirin and surveillance colonoscopy is cost-effective in secondary prevention of colorectal cancer in individuals with advanced adenomas: network meta-analysis and cost-effectiveness analysis
Source: BMC Gastroenterol. 2021 Mar 20;21:130. doi: 10.1186/s12876-021-01715-7 (PMC7981989; doi:10.1186/s12876-021-01715-7)
Supplement: Supplementary file 1 — Additional file 1. Supporting information. [file 12876_2021_1715_MOESM1_ESM.docx]

**Supporting information (Online publication only)**

**Very-Low-Dose Aspirin and Surveillance Colonoscopy is Cost-effective in Secondary Prevention of Colorectal Cancer in Individuals with Advanced Adenomas: Network Meta-analysis and Cost-effectiveness Analysis**

**S 1.1 Definitions of efficacy and safety outcomes**

Primary outcome was the incidence of recurrent advanced colorectal adenomas (ACAs) following chemo-preventive agent alone or in combination with surveillance colonoscopy. Available data suggest that the progression of adenomas to colorectal cancer (adenoma-carcinoma sequence) is driven by a multistep process of specific genetical changes. More than 85% of CRCs are assumed to have developed in an age-dependent manner from advanced adenomas with an estimated annual transition probability of 2.6% to 5.2%. Hence, adenomas, especially advanced adenomas should be resected (polypectomy) entirely to prevent the progression of CRC. However, even with complete resection, the recurrence rate is still relatively high. Hence, the recurrence of advanced adenomas is considered as an important surrogate endpoint in chemoprevention trials among individuals with a history of colorectal adenomas.

In our study, ACA was defined by one or more of the following features: 1 cm or larger, with villous or tubulovillous histology, with high-grade dysplasia, and/or with intra-mucosal carcinoma or invasive cancer^1^.

Serious adverse events (SAEs) were defined as events resulting in death, hospital admission because of an adverse event, severe gastrointestinal (GI) bleeding, cardiovascular (CV) or non-CV complications, or discontinuation of intervention due to an adverse event or events that were defined as serious or severe by the study authors.

Serious CV events were defined as composite of CV death, myocardial infarction, stroke, heart failure, thromboembolic event, or defined as serious CV event by the study investigators^2^.

**S 1.2 Search strategy and study selection**

**S Table 1.2.1 Search strategy**

| Search algorithms designed for Medline database | |
| --- | --- |
| #1 | Adenoma |
| #2 | Adenoma$ |
| #3 | Adenocarcinoma |
| #4 | Adenomatous$ |
| #5 | Adenomatous polyps |
| #6 | Colon cancer$ |
| #7 | Colon neoplas$ |
| #8 | Colon tumo$ |
| #9 | Colonic cancer$ |
| #10 | Colonic neoplas$ |
| #11 | Colonic neoplasms |
| #12 | Colonic polyps |
| #13 | Colonic tumo$ |
| #14 | Colorectal cancer$ |
| #15 | Colorectal neoplas$ |
| #16 | Colorectal neoplasms |
| #17 | Colorectal tumo$ |
| #18 | Intestinal polyps |
| #19 | Polyp$ |
| #20 | Rectal cancer$ |
| #21 | Rectal neoplas$ |
| #22 | Rectal neoplasms |
| #23 | Rectal tumo$ |
| #24 | Rectum cancer$ |
| #25 | Rectum neoplas$ |
| #26 | Rectum tumo$ |
| #27 | #1 OR #2 OR #3 OR #4 OR #5 OR #6 OR #7 OR #8 OR #9 OR #10 OR #11 OR #12 OR #13 OR #14 OR #15 OR #16 OR #17 OR #18 OR #19 OR #20 OR #21 OR #22 OR #23 OR #24 OR #25 OR #26 |
| #28 | Aspirin |
| #29 | Acetylsalicylic acid |
| #30 | COX-1 inhibitor$ |
| #31 | COX-2 inhibitor$ |
| #32 | COX-2 selective inhibitor$ |
| #33 | Coxib$ |
| #34 | Cyclooxygenase 1 inhibitor$ |
| #35 | Cyclooxygenase 2 inhibitor$ |
| #36 | Cyclooxygenase 2 inhibitors |
| #37 | Cyclooxygenase inhibitor$ |
| #38 | Cyclo-oxygenase inhibitor$ |
| #39 | Cyclooxygenase inhibitors |
| #40 | Nonsteroidal antiinflammatory$ |
| #41 | Non-steroidal antiinflammatory$ |
| #42 | Nonsteroidal anti-inflammatory$ |
| #43 | Non-steroidal anti-inflammatory$ |
| #44 | Anti-inflammatory agents, non-steroidal |
| #45 | NSAID$ |
| #46 | #28 OR #29 OR #30 OR #31 OR #32 OR #33 OR #34 OR #35 OR #36 OR #37 OR #38 OR #39 OR #40 OR #41 OR #42 OR #43 OR #44 OR #45 |
| #47 | Folate$ |
| #48 | Folic$ |
| #49 | Folic acid |
| #50 | #47 OR #48 OR #49 |
| #51 | Calcium |
| #52 | Calcium$ |
| #53 | Calcium, dietary |
| #54 | #51 OR #52 OR #53 |
| #55 | Cholecalciferol |
| #56 | Cholecalciferol$ |
| #57 | Ergocalciferol$ |
| #58 | Ergocalciferols |
| #59 | Vitamin D |
| #60 | #55 OR #56 OR #57 OR #58 OR #59 |
| #61 | Antioxidant$ |
| #62 | Anti-oxidant$ |
| #63 | Antioxidants |
| #64 | Ascorbic acid |
| #65 | Vitamin C |
| #66 | Vitamin A |
| #67 | Beta-carotene |
| #68 | Carotenoid$ |
| #69 | Carotenoids |
| #70 | Selenium |
| #71 | Tocopherol$ |
| #72 | Tocopherols |
| #73 | Tocotrienol$ |
| #74 | Tocotrienols |
| #75 | Alpha-tocopherol$ |
| #76 | Vitamin E |
| #77 | #61 OR #62 OR #63 OR #64 OR #65 OR #66 OR #67 OR #68 OR #69 OR #70 OR #71 OR #72 OR #73 OR #74 OR #75 OR #76 |
| #78 | Clinical trial |
| #79 | Controlled clinical trial |
| #80 | Single blind method |
| #81 | Double blind method |
| #82 | Placebo |
| #83 | Placebo$ |
| #84 | Random$ |
| #85 | Random allocation |
| #86 | Randomised controlled trial |
| #87 | Randomised controlled trials |
| #88 | #78 OR #79 OR #80 OR #81 OR #82 OR #83 OR #84 OR #85 OR #86 OR #87 |
| #89 | #46 OR #50 OR #54 OR #60 OR #77 |
| #90 | #27 AND #88 AND #89 |
| This search strategy (include search terms for both adenomas and colorectal cancer (CRC)) was developed for the whole study. | |

**S Figure 1.2.1 PRISMA flow diagram**

**
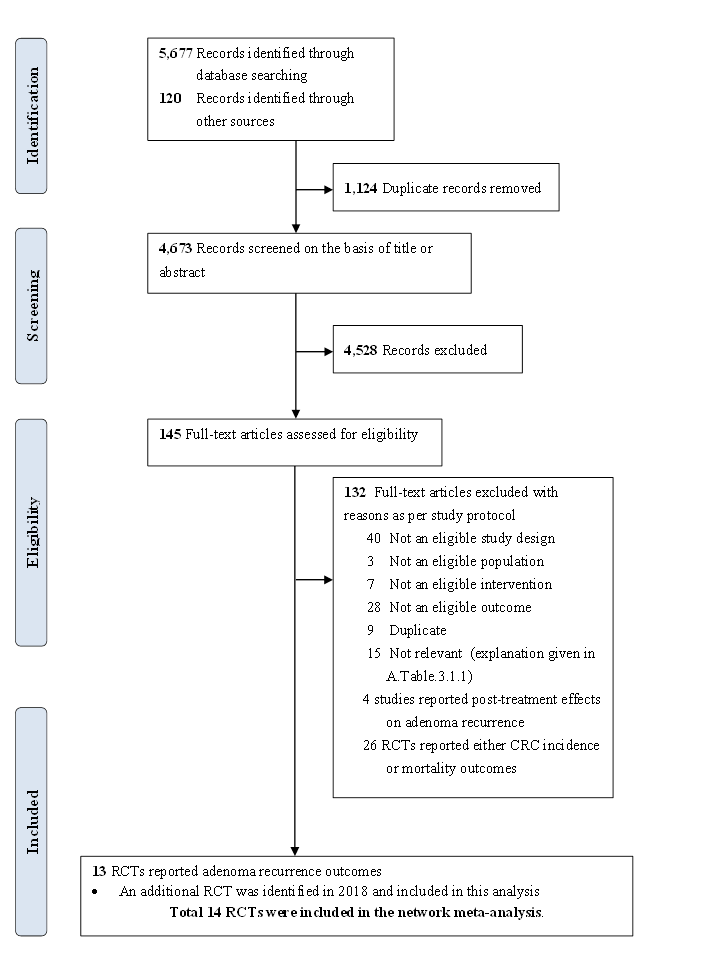
**

**S 1.3 Inclusion criteria**

Studies included were RCTs with a treatment duration of at least one year and met the following inclusion criteria: participants were adults (age ≥18 years) at increased risk of developing CRC due to the previous history of adenomas, previous polypectomy and with a documented clear colon before randomisation; intervention with any chemo-preventive agent (CPA) (aspirin at different doses, non-steroidal anti-inflammatory drugs (NSAIDs) including celecoxib at different doses, calcium, and vitamin D) alone or in combination with surveillance colonoscopy and comparator was another CPA or placebo; and the outcome was the proportion of subjects who developed advanced colorectal adenomas.

Individuals with a history of familial adenomatous polyposis (FAP) or Lynch syndrome were not included in analysis. Likewise, those RCTs that recruited subjects before 1990 or where the baseline and/or follow-up colonoscopy examinations were not reported available^3^. Among the NSAIDs, rofecoxib and valdecoxib have been withdrawn from the market^4^; and therefore also excluded from the analysis. Aspirin was classified into three groups based on dose as described by the latest report of USPSTF ^5^: high-dose or ASAHD (> 325 mg/day), low-dose or ASALD (> 100 and ≤ 325 mg/day) and very-low-dose or ASAVLD (≤ 100 mg/day) aspirin. Similarly, celecoxib was classified based on dose into two groups: celecoxib 400 mg/day and 800 mg/day (as reported by RCTs). Calcium was not grouped into different doses due to heterogeneity in doses reported from available trials. Folic acid as a CPA was not included in this analysis due to two reasons: 1) no evidence of efficacy against adenoma recurrence^6^; and 2) increased risk of sessile serrated adenomas in a recent long-term follow-up of RCT^7^.

***List of excluded RCTs with reasons:***

| **Authors**  **(citation)** | **Reasons for exclusion** |
| --- | --- |
| Ladenheim et al. ^8^ | Randomised placebo-controlled trial.  Interventions (number of participants): sulindac 300mg/day (n=44); placebo (n=40).  Primary outcome was percentage of patients for whom all polyps either disappeared or regressed. Incidence of recurrent adenomas was not reported. |
| Baron et al. ^9^ | Duplicate of Baron et al., 1999 study. ^10^ |
| Benamouzig et al.^11^ | The latest 4-year results of this study were available; hence, the latest results from Benamouzig et al., 2012 were used for the analysis ^12^. |
| Baron et al. ^13^ | Randomised, double-blind, placebo-controlled trial.  Interventions and participants: rofecoxib 25 mg/day (n=1,277); Placebo (n=1,293).  Rofecoxib was withdrawn from the market and therefore excluded. |
| Jaszewski et al. ^14^ | Did not report event data (only mean number of adenoma). |
| Benamouzig et al. ^15^ | Duplicate of Benamouzig et al. 2012. ^12^ |
| Li et al. ^16^ | This study was identified from the reference list of Zhao et al.^17^ As per the author’s description, this paper was retrieved from “Chinese biomedical literature service system (SinoMed) database, a study written in the Chinese language and was graded as low quality”. Authors of the present study have searched the “Chinese biomedical literature service system (SinoMed) database” to retrieve this article. Unfortunately, the authors were not able to identify the full text of this study. Hence it was excluded from the analysis. |
| Takayama et al. ^18^ | Duration of treatment was only 2 months and follow-up was approximately 1 year. Interventions (number of participants): sulindac 300 mg/day (n=60); etodolac 400 mg/day (n=61); placebo (n=58). |
| Bonelli et al. ^19^ | Trial recruited participants before 1990. |
| McKeown-Eyssenet al. ^20^ | Trial recruited participants before 1990. |
| Roncucci et al. ^21^ | Trial recruited participants before 1990. |
| Greenberg et al. ^22^ | Trial recruited participants before 1990. |
| MacLennan et al. ^23^ | Trial recruited participants before 1990. |
| Ponz de Leon et al. ^24^ | Status of colon post-polypectomy was not clear. |
| Hofstad et al. ^25^ | No complete polypectomy, some polyps were left in situ. |
| Wu et al. ^26^ | Intervention was folic acid. Hence excluded. |
| Cole et al. ^27^ | Factorial trial with folic acid (excluded from analysis) and aspirin as interventions. Only the results of aspirin arm were used in this analysis that reported in an earlier paper by Baron et al. (2003) ^28^. |
| Meyskens et al. ^29^ | Intervention was a combination of sulindac and difluoromethylornithine (DFMO). DFMO is not considered a CPA for CRC. Hence efficacy from this trial not represented the effect of NSAID alone. Furthermore, DFMO is not available in our setting. |

**S 1.4 Description of data extraction and quality assessment**

Requisite data on the study, participants, and treatment-related characteristics were extracted independently and in duplicate by two reviewers (SKV, SK) into a data extraction form, and discrepancies were resolved by another reviewer (NC) after group discussion. If multiple publications of the same trial were retrieved, only the most recent, informative or relevant data were included. Data on efficacy outcomes were extracted using modified intention-to-treat analysis (that is, subjects who received at least one dose of CPA at any strength and had at least one colonoscopy after randomisation). Data on safety outcomes were extracted by the same intention-to-treat principle, allocating the initial number of randomised participants to each trial arm. Participants who were lost to follow-up were considered free of adverse events. For a trial with a factorial design which comprised of an inappropriate intervention (an intervention not defined as CPA in this study and included in factorial comparison) or did not report the outcome for individuals arms, then the data was extracted based on ‘at-margins’ analysis principle ^30^ i.e. comparing all groups that received a relevant CPA with groups that did not receive that particular CPA.

The risk of bias (RoB) within each trial that reported efficacy outcomes was assessed by using the revised Cochrane risk of bias tool (RoB 2.0) ^31^. The RoB version 2.0 assessment tool for RCT have five domains as follows: 1) bias due to randomisation process; 2) bias due to deviations from intended interventions; 3) bias due to missing outcome data; 4) bias in measurement of the outcome; and 5) bias in the selection of the reported results. The response options for an overall risk of bias judgment of a trial were categorised as low ROB, some concerns or high ROB based on five domain-level judgments. Two reviewers independently assessed (SKV, LKG) the ROB within each study. Reviewers resolved disagreements by discussion, and one of two arbitrators adjudicated any unsolved disagreements.

**S 1.5 Network meta-analysis: statistical methods**

The relative intervention effect was expressed in risk ratio (RR) and presented along with 95% confidence interval (CI). Risk ratio is the ratio between the incidence of recurrent colorectal advanced adenomas (or any recurrent adenomas or serious adverse events) in the intervention arm to that in the placebo or control arm. A risk ratio below one indicates that the treatment was associated with a lower risk of the outcome than the comparator while a RR above one indicates that the treatment was associated with a greater risk of the outcome than the comparator.

For direct comparisons, a standard pairwise meta-analysis was performed by using a random-effects model (The DerSimonian-Laird method) ^32^. If a direct comparison was based on two or more studies, heterogeneity between trials was assessed by considering the I^2^ statistics; an I^2^ estimate ≥ 50% was interpreted as evidence of substantial levels of heterogeneity ^33^. A random-effects NMA using the consistency model was applied in comparison of all interventions using direct and indirect data ^34,35^. Network inconsistency assumption, which refers to a disagreement between the direct and indirect estimates, was evaluated using global inconsistency test by fitting design-by-treatment in the inconsistency model ^36,37^. Placebo was used as the common comparator in the network model. To rank the intervention hierarchy in NMA, the surface under the cumulative ranking (SUCRA) curves were estimated. Higher SUCRA scores (ranging from 0 to 1) correspond to a higher ranking for prevention of recurrent advanced adenomas and higher ranking for safety in terms of serious adverse events. Publication bias was examined with a comparison-adjusted funnel plot ^38^. To assess the robustness of the findings of primary efficacy outcomes, multiples sensitivity analyses based on the following assumptions were performed: restricting studies with low-risk of bias; excluding those RCTs with a population with a history of CRC and trials with duration of follow-up of less than two years.

The quality of evidence from NMA was evaluated by the Grading of Recommendations, Assessment, Development and Evaluation (GRADE) approach ^39^. In this approach, the evaluation typically starts with direct evidence using GRADEpro version 3.6.1 (web based software, McMaster University, 2014). Generally, the direct evidence from RCTs is often rated as high quality but could be rated down based on the ROB, indirectness, imprecision, inconsistency (commonly known as heterogeneity), and/or publication bias to levels of moderate, low, and very-low quality. The quality rating of indirect estimates starts at the lowermost rating of the two direct estimates that contribute to the indirect estimate of the comparison of interest as first-order loops. In the presence of intransitivity (typically defined as the differences in study characteristics that may modify treatment effect of direct comparison), the indirect estimate could be rated down from the lower value of the confidence ratings of the contributing direct comparisons. Finally, if both direct and indirect estimates are available and are similar in values (known as coherence) then the higher of the two quality ratings can be assigned to the quality rating for NMA estimates.

**S 1.5.1 Trial sequential analysis**

When a pairwise meta-analysis comprises a small number of RCTs and subjects, random errors can lead to deceptive conclusions ^40,41^. Some ‘positive’ meta-analytic results may be due to the play of chance (random error) rather than due to an underlying ‘true’ intervention effect. Trial sequential analysis (TSA) considers the risks of random errors and demonstrates the required sample size and boundaries that consider whether the evidence in a pairwise meta-analysis is conclusive ^41^. If only a few RCTs and hence less number of patients included in the pairwise meta-analyses of many comparisons, then trial sequential analyses are performed using the TSA software package (available at <http://www.ctu.dk>) ^42^. TSA combines information size estimation for meta-analysis (cumulated sample size of included trials) with an adjusted threshold for statistical significance in the cumulative meta-analysis. In our analysis, TSA was performed only for those CPAs with evidence of efficacy documented in a pairwise comparison of at least 2 RCTs.

**S 1.5.2 Risk-benefit integrated analysis**

In our study, the risk-benefit integrated analysis was used to review the potential benefits (prevention of recurrent advanced adenomas) and risks (serious adverse events) of CPAs that demonstrated evidence of efficacy in preventing advanced adenoma recurrence and was performed according to Dulai S et al ^43^. To estimate the absolute risk of advanced adenomas with interventions, risk ratios derived from the placebo comparisons of CPAs in the NMA were used. To estimate control risk, population-level risks of advanced adenomas derived from the published pooled estimates of the National Cancer Institute pooling project was used ^44^. To understand the potential risks, the pooled risk of serious adverse events in the placebo group (estimated using the metaprop command in STATA^45^) as a measure of baseline risk was estimated. Subsequently, risk ratios derived from the placebo comparisons of each CPA in NMA for serious adverse events were used to estimate the absolute risk associated with that particular intervention. Subsequently, the excess benefit and risk of serious adverse events (over placebo) per 1,000 individuals who received the intervention were presented. Estimates of absolute risk were generated with the GRADEpro version 3.6.1 (McMaster University, 2014) ^46^.

**S 2.1 Description of model and its assumptions**

A cost-effectiveness analysis was performed to determine the total costs and benefits associated with the use of ASAVLD alone or in combination with routine surveillance colonoscopy (every 3 years) compared to no screening or surveillance colonoscopy for CRC prevention in individuals with a history of advanced colorectal adenomas.

A previously published Markov model Tappenden et al. ^47^ in 2003 to assess the cost-effectiveness of different options for CRC screening had been modified to suit our study by incorporating the expected costs and outcomes of intervention. Due to insufficient evidence regarding the rate at which *de novo* CRCs could develop, the model presented in our study had assumed that all CRCs were developed from preceding adenomas. This model was comprised of three inter-related components: a state transition model which simulate the natural history of CRC; a model of surveillance screening and chemoprevention which directly interact with the natural history model; and a model of mortality.

The primary research question of this analysis addressed the following: is the use of ASAVLD alone or in combination with routine surveillance colonoscopy cost-effective as compared to no surveillance or routine surveillance colonoscopy in a population at increased risk due to a personal history of advanced colorectal adenomas in Malaysia?

The basic structure of the Markov model is outlined in S Figure 2.1.

***S Figure 2.1 The basic structure of the model***


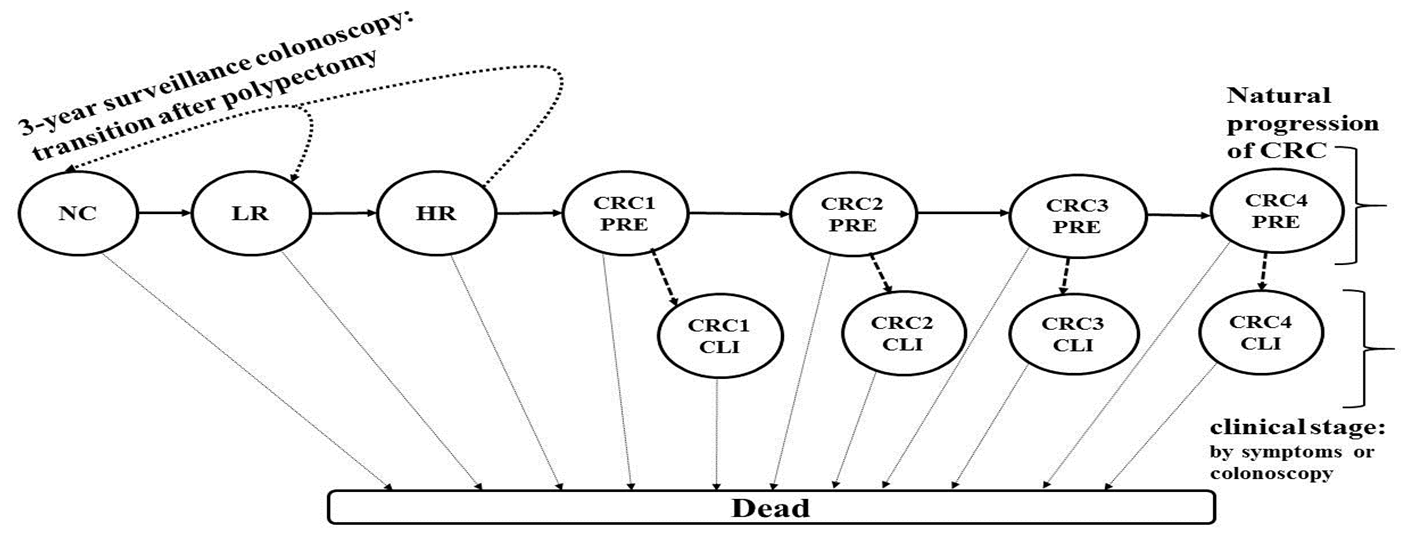


***Abbreviations:*** *CLI, clinical stage; CRC, colorectal cancer; HR, high-risk state; LR, low-risk state; NC, normal colon; PRE, pre-clinical stage*

*Note: From high-risk state, backward-transition can occur in both LR state and NC and it depends on the diagnostic accuracy of colonoscopy*

**S 2.2 Detailed descriptions of population and intervention under assessment**

| **Appendix Table 2.2.1 Description of the population under assessment** | | |
| --- | --- | --- |
| Definition | | The population is defined as individuals at increased risk of CRC due to a personal history of advanced colorectal adenomas of the colon/rectum at initial screening colonoscopy and undergo polypectomy at 50 years of age and reported clean colon/rectum. |
| Definition of advanced adenomas in the population under assessment | | Advanced colorectal adenomas (typically considered as high-risk adenomas) are usually defined in randomised controlled trials (RCTs) as an adenoma with high-grade dysplasia, >10 mm in size, or a villous (or tubulovillous) features ^1^. |
| Decision concerning the choice of population for assessment | | Individuals with a history of low-risk adenomas (that is, typically non-advanced adenomas) were not included in this analysis due to less favourable risk-benefit profile with ASAVLD as demonstrated in our NMA. Moreover, the assumption was that only advanced colorectal adenomas are at higher risks of progressing into CRC ^48^. |
| Reasons for excluding average-risk individuals | | The use of very-low-dose aspirin (ASAVLD) for primary prevention of CRC in individuals at average risk was excluded from the economic analysis presented here. This exclusion is justified as:  1) the data on the dose-specific effects of aspirin for primary prevention of CRC is still limited to make a definitive conclusion;  2) the CRC incidence rate in Malaysia (the lifetime risk of developing CRC in the general population is below 6% ^49^) is low compared to Western countries who adopted several primary prevention strategies ^50^;  3) ASAVLD is not even routinely recommended for primary prevention of cardiovascular (CV) disease in Malaysia due to its risks ^51^. |
| Reasons for excluding high-risk individuals | | Individuals at high risk of CRC due to familial adenomatous polyposis (FAP) or hereditary nonpolyposis colorectal cancer (HNPCC) or due to a family history of CRC were not included in the analysis because of following reasons:  1) there is limited evidence concerning the impact of reducing adenoma recurrence using chemoprevention in these patients ^52^  2) little is known about the underlying disease natural history of FAP and HNPCC patients |
| **Appendix Table 2.2.2 Description of intervention under assessment** | | |
| Recommendations for colonoscopy surveillance | Recommendations from the U.S. Multi-Society Task Force on Colorectal Cancer^53^ for colonoscopy surveillance in individuals with a history of advanced adenomas: one or more tubular adenomas ≥1 cm: every 3 years (high quality of evidence); one or more villous adenomas: every 3 years (moderate quality of evidence); adenoma with high-grade dysplasia: every 3 years (moderate quality of evidence); similarly, the published Clinical Practice Guideline on Management of Colorectal Carcinoma in Malaysia also recommends every 3 years of surveillance ^54^. As per recommendation, the routine surveillance colonoscopy in study population under assessment was every 3 years. | |
| Starting and completion age of surveillance colonoscopy in population | According to the United States Preventive Services Task Force (USPSTF) 2016 recommendation, screening for CRC in average-risk adults should be started at the age of 50 years, hence we have used 50 years old as the starting age for prevention benefit in our model ^55^.  For patients aged 75–85 years, the US Multi-Society Task Force on Colorectal Cancer recommended screening based on comorbidities and findings of preceding colonoscopy ^53^. Hence, our model assumed a surveillance colonoscopy until the age of 75 years as per average life expectancy of Malaysian population ^56^ and followed the study population until remaining lifetime. | |
| Decision concerning the choice of ASAVLD | Conclusion of NMA results:   1. ASAVLD has demonstrated a greater reduction in recurrent advanced adenomas. 2. The risk-benefit profile favours the use of ASAVLD, especially in those with a history of high-risk adenomas. 3. ASAVLD was found superior to ASALD and similar to celecoxib (at different doses) when comparative efficacy of CPAs is assessed. 4. The evidence is of moderate quality and conclusive. 5. The observed protective effect does reduce over time after treatment cessation. | |
| Reasons for excluding other interventions | Other potential CPAs including low-dose aspirin (>100-325 mg/day), high-dose aspirin (>325 mg/day), calcium, celecoxib and other non-steroidal anti-inflammatory drugs (NSAIDs) were excluded from analysis either because of insufficient evidence to describe effectiveness against recurrent colorectal adenomas or due to its potential harms. For example although evidence supports the effectiveness of celecoxib in prevention of recurrent colorectal adenomas, however from recent meta-analyses, it was ranked low in term of safety among other CPAs and there were reports of significant serious CV events ^57,58^. Moreover, no effect on adenoma recurrence was noticed after discontinuing celecoxib for more than 2 years ^57^. Similarly, the evidence surrounding the use of low-high dose aspirin and calcium for chemoprevention has been mixed (NMA results). The results of some of these RCTs have been inconsistent and the accumulated evidence from these RCTs is inconclusive^59,60^. | |

**S 2.3 Definitions of health states**

Our model simulated a series of transitions of study cohort based on yearly probabilities occurring sequentially between the following 12 mutually exclusive health states: normal colon, low-risk state, high-risk state, CRC stage I-IV (pre-clinical), CRC stage I-IV (clinical), and death. Non-advanced adenomas (for example, tubular adenomas <1 cm in diameter) were considered low-risk state ^53^. Advanced adenomas (definition provided in Appendix Table 2.2.1) were considered high-risk state. A subject diagnosed with CRC and started on cancer treatment was considered as clinical stage; and an undiagnosed subject was considered as pre-clinical stage. Cancer stage was modelled according to the American Joint Committee on Cancer (AJCC) manual ^61^ and classified as stage I–II (assumed to be local), stage III (regional) and stage IV (distant) ^62^.

**S 3.1 Transition Probability for normal colon to low-risk state**

| Risk of new non-advanced adenomas (low-risk adenomas) obtained from pooled individual data of 8 prospective studies comprising 4523 men and women with a previous history of high-risk adenomas (advanced adenomas) ^44^ was 35.3% (95%CI, 33.9–36.7) after 2 years. | |
| --- | --- |
| Annual probability was derived from the following method ^63,64^: The 2-year probability of non-advanced adenomas was 35.3%. The incidence rate was assumed to be constant for over 2 years. | |
| Rate = -ln (1-p)/t | = ln (1-0.353)/2 **=**0.2177 |
| Probability= 1- exp (-rt) | = 1-exp (-0.2177x1)= 0.1956 |
| Lower and upper limits of 95% CI were converted similarly.  Annual probability = 0.1956 (95% CI, 0.1870 to 0.2044). | |
| To obtain standard errors from confidence intervals ^32^:  Standard error (SE) = (upper limit – lower limit) / 3.92  Annual probability =0.1956 (SE 0.0044) | |
| Abbreviations: p=probability; t=time; r=rate | |

**S 3.2 Transition Probability to high-risk state**

The following are four baseline characteristics of high risk adenomas as reported in the National Cancer Institute pooling project ^44^: history of adenomas with 1) high-grade dysplasia; 2) villous components (villous/tubulo-villous); 3) size more than 1 cm; and 4) size more than 2 cm. Advanced adenomas are typically defined as adenomas >10 mm, villous components (villous/tubulo-villous), or with high-grade/severe dysplasia. Evaluation of high-risk state as a subgroup population was performed at 2-year follow-up were then meta-analysed.


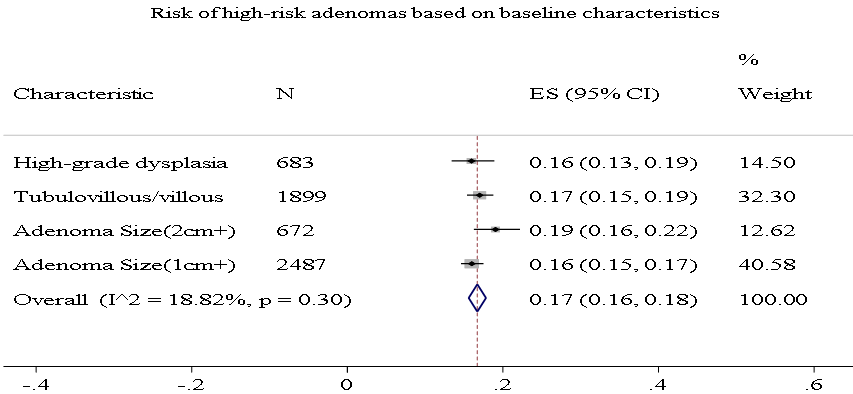


Annual transition probability was calculated using method mentioned in Appendix Table 4.3.1. 0.0890 (95% CI 0.0834 to 0.0945) =0.0890 (SE 0.0028)

Abbreviations: ES, effect size; N, number of subjects in each subgroup

**S 3.3 Transition Probability to stage 1 pre-clinical CRC state**

A birth cohort analysis has reported transition rates from the carriage of advanced adenoma to carriage of early stage CRC by sex and age among 3,593,420 participants in the German screening colonoscopy program in 2003–2010.^65^ This study has demonstrated a similar (not statistically significant) transition rates from advanced adenoma to CRC carriage in men and women. However, the risk of CRC significantly increased with age ^65^.

Previous analysis (Brenner H 2007) also reported age and sex-specific estimates of transition rates from advanced adenomas to CRC by combining the data of a nationwide screening colonoscopy registry and national data on CRC incidence from the same population (2003 and 2004 (n = 840,149)).

| **Appendix Table 3.3.1** | | | |
| --- | --- | --- | --- |
| **Age** | **Annual transition rate (%)** | **Annual transition probability** | **Comments/reference** |
| 50-54 | NA | NA | Assumed to be 0.2600 |
| 55-59 | 2.60% | 0.2600 | Brenner H 2013 |
| 60-64 | 3.10% | 0.3100 | Brenner H 2013 |
| 65-69 | 4.15% | 0.4150 | Brenner H 2013 |
| 70-74 | 4.60% | 0.4600 | Brenner H 2013 |
| 75-79 | 5.20% | 0.5200 | Brenner H 2013 |
| 80-84 | 5.35% | 0.5350 | Brenner H 2007 |
| 85+ | 5.35% | 0.5350 | Brenner H 2007 |
| NA: not available | | | |

**S 3.4 Per-patient miss rate**

The impact of surveillance colonoscopy on adenomas was evaluated in our analysis based on the number of subjects in the cohort redistributed back to the normal colon from low and high-risk states (note: from high risk state, proportion of patients redistributed to both low risk state and normal state depends on the diagnostic accuracy of colonoscopy). The probability of aforementioned transitions was derived from per-patient miss rate (it is different from adenoma miss rate), a unique parameter calculated from per-patient analysis in recent prospective studies (characteristics of studies provided in Appendix Table 3.4.1), which is used to describe the effectiveness of colonoscopy to detect and remove all adenomas (polypectomy) and report as clean colon as assumed in clinical trials. Per-patient miss rate resembles the outcome assessment in chemoprevention clinical trials in individuals with history of colorectal adenomas (that is, these trials assessed the number of patients with and without at least one adenoma observed during surveillance colonoscopy) and allows measures to rationalizse rationalise impact of study intervention. A per-patient miss rate was calculated as the number of patients with at least one neoplastic adenoma missed by the surveillance colonoscopy divided by the total number of patients with at least one neoplastic adenoma at baseline ^66^ *[per-patient miss rate = the number of patients with missing adenoma/total number of patients with at least one neoplastic adenoma undergo surveillance]*.

Therefore, the rate of patients redistributed back to the normal colon = 1- per-patient miss rate (for example, assuming 1,000 individuals with baseline low-risk adenoma underwent surveillance colonoscopy, and if the per-patient missing rate for low-risk adenoma is 10% means, 100 individuals would be retained in the low-risk adenoma state and 900 patients would be returned to normal colon state after polypectomy). Colonoscopy is usually considered the gold standard to detect colorectal adenomas. However, variability in sensitivity of this technique to detect adenomas have been observed in different studies/countries (due to variation in technology, endoscopist expertise, pre-colonoscopy preparations, etc.). Hence, per-patient miss rates for low-risk and high-risk adenomas were reasonably estimated from meta-analyses using data from the most recent studies (after 2010) conducted in Asian population using different techniques for evaluation of colon (study characteristics provided in Appendix Table 3.4.1).

**S Table 3.4.1 Characteristics of studies from Asian countries reported per-patient miss rate**

| **Author (reference)** | **Year** | **Country** | **Colonoscopy technique** | **Bowel cleansing** | **Quality of bowel preparation**  **Aronchick scale** ^67^ |
| --- | --- | --- | --- | --- | --- |
| Kim NH ^68^ | 2017 | Korea | Conventional colonoscopes (CF-Q260AI and CF-H260AI; Olympus Medical Systems, Tokyo, Japan | 4 L of PEG (Taejoon Pharm, Seoul, Korea) | Excellent for 24.2%, good for 63.5%, and fair for 12.3% |
| Shin JG ^69^ | 2017 | Korea | Conventional and cap-assisted colonoscopy | NA | NA |
| Chang JY ^70^ | 2018 | Korea | Standard colonoscopes (CF Q240, CF Q260, CF H260; Olympus Optical Co., Ltd., Tokyo, Japan) with EVIS LUCERA system (Olympus, Tokyo, Japan) | 4L PEG (72.6%); SPMO (13.2%); 2L PEG + AA (12.2%); Others (2%) | Defined as inadequate |
| Li Xiang ^71^ | 2014 | China | Electronic colonoscopies CF-240 I and CF-260 I (Olympus, Tokyo, Japan) | PEG, sodium phosphate or mannitol | Unclear |
| Hong SN ^72^ | 2012 | Korea | Tandem high-definition CF-H260AI colonoscope (Olympus, Tokyo, Japan) | 4L PEG | Excellent for 32%, good for 41%, fair for 20% and poor for 6% |
| 4L PEG= 4 litre of polyethylene glycol; SPMO= Sodium picosulfate + magnesium oxide; AA=ascorbic acid | | | | | |

**S Figure 3.4.1 Meta-analysis of per-patient miss rate for low-risk adenomas**

**
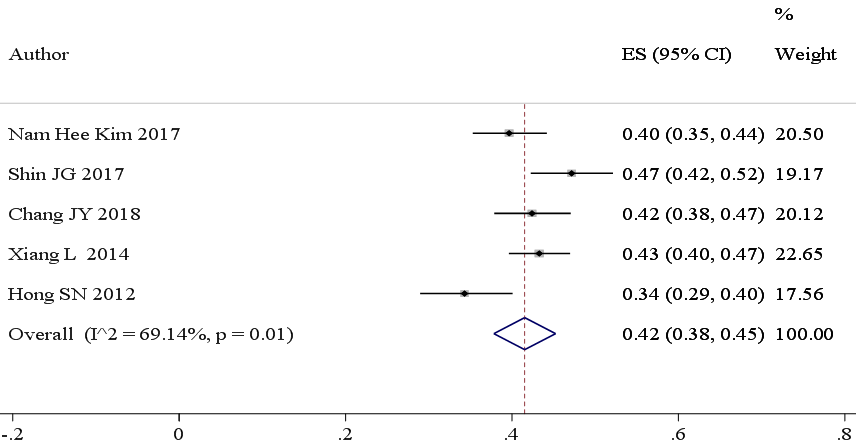
**

Transition probability from low risk adenoma to normal colon: 0.58 (95% CI 0.55 to 0.62) based on meta-analysis of studies from Asian countries, as shown in Appendix Table 3.4.1.

**S Figure 3.4.2 Meta-analysis of per-patient miss rate for high-risk adenomas**

**
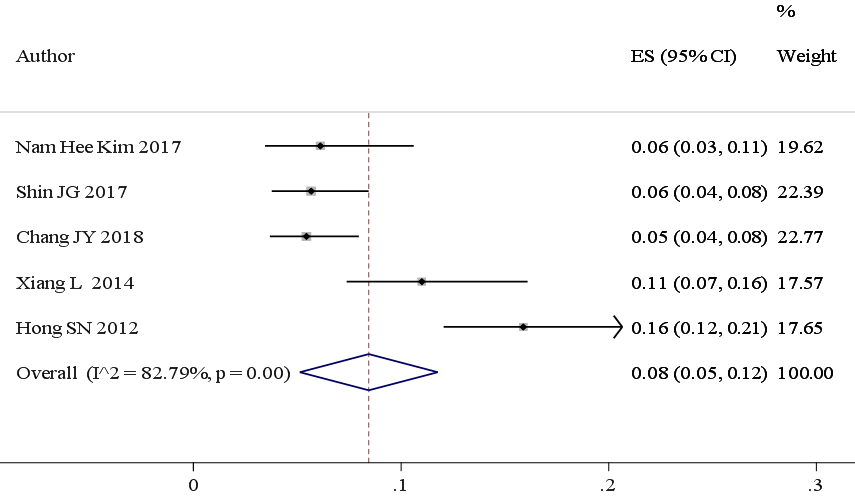
**

Transition probability from high-risk adenoma to normal colon/ LR state: 0.92 (95% CI 0.88 to 0.95) based on meta-analysis of studies from Asian countries as shown in Appendix Table 3.4.1.

Transition probability to normal colon incorporated into the model equation based on the per-patient miss rate of low and high risk polyps.

**S 3.5 Sensitivity of colonoscopy on CRC**

The sensitivity of colonoscopy for detection of CRC was obtained from a recent systematic review and meta-analysis, including 25 studies and 9223 patients. The overall sensitivity was 94.7% (178 of 188; 95% CI: 90.4%, 97.2%) ^73^. The sensitivity of colonoscopy for late stage cancers (CRC2 and CRC3) was taken from an economic evaluation conducted for the National Institute for Health Research (NIHR) ^52^.

**S 3.6 Model Parameters for chemoprevention**

Based on two RCTs, ASAVLD has demonstrated efficacy for chemoprevention in recurrent advanced adenomas:

Baron 2003 ^28^ (The Aspirin/Folate Polyp Prevention study): United States

Ishikawa 2014 ^74^: Japan

Characteristics of studies were provided in appendix tables Table 4.1.1-2.

It was assumed that a constant relative risk could be applied over time to the transition probability from the normal colon to low-risk state and from low-risk state to high-risk state while the subject is on chemoprevention.

**S Figure 3.6.1 Relative risk of recurrent advanced adenomas (ASAVLD versus placebo)**


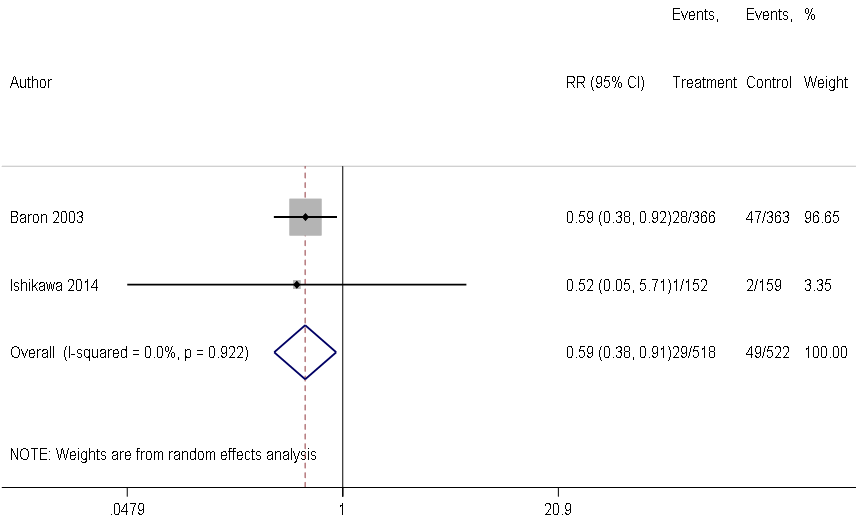


A meta-analysis using random random-effects model demonstrated a statistically significant risk reduction of 41% of recurrent advanced adenomas (relative risk (RR) 0.59 (95% CI, 0.38 to 0.91).

**S Figure 3.6.2 Relative risk of recurrent non-advanced adenomas (ASAVLD versus placebo)**

For the included RCTs of ASAVLD in individuals at increased risk ^28,74^, the number of subjects with recurrent low-risk adenomas identified during follow-up colonoscopy was not reported. However, the number of subjects with any adenomas and advanced adenomas (that is, high-risk adenomas) was provided. Hence, the number of subjects with low-risk adenomas was estimated as the difference between the number of subjects with any adenomas and advanced adenomas.


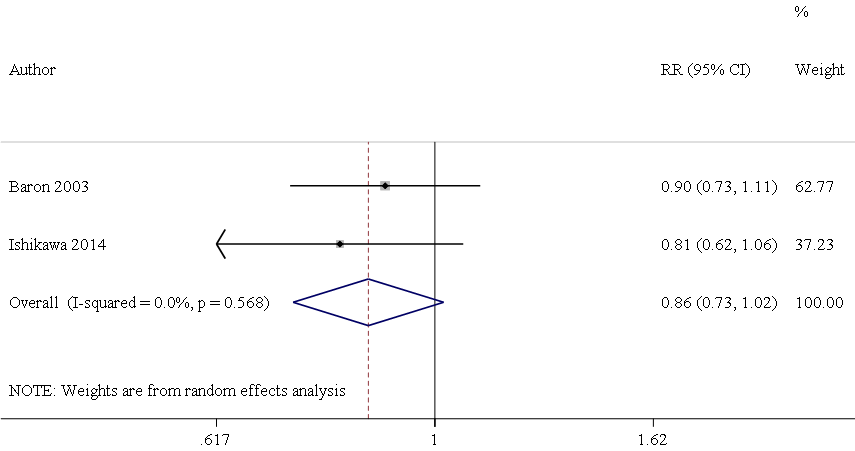


A meta-analysis using random effects model demonstrated a statistically significant risk reduction of 14% of recurrent non-advanced adenomas (Relative risk (RR) 0.86 (95% CI, 0.73 to 1.02).

**S 3.7 Model Parameters for chemoprevention related harms**

**S 3.7.1 Major bleeding (any) due to ASAVLD per year**

Individuals receiving aspirin were anticipated to develop major bleeding events every year that would impede further ASAVLD (≤ 100 mg/day) chemoprevention. If these individuals survived, they were similarly transitioned to colonoscopy surveillance. To identify the probability of major bleeding events per year the method derived from a recent meta-analysis of nine primary prevention trials ^75^ was used:

Mean follow-up: 6.6 years; number of participants: 157,248; mean age: 61.3 years (SD = 2.2 years); relative risk (RR) of major bleeding events due to ASAVLD=1.45 (95% CI, 1.28 to 1.65). Probability was derived from the following formula: Probability (ASVLD) = RR x Probability in unexposed group ^63,64^

=1.45 x 0.0099 = 0.01435= 1.44% *over the entire study period*

*Probability of major bleeding events per year was calculated using method mentioned in Appendix table 4.3.1.* =0.0022 (SE 0.0005)

**S 3.7.2 Annual probabilities of harms due to ASAVLD**

Only excess harms to those normally experienced within the population were included in our analysis. The RR of experiencing each harm (major GI bleeding, peptic ulcer and dyspepsia) is applied to the baseline incidence within the general population to calculate the additional probability of experiencing each harm as a result of ASAVLD therapy.

The anticipated baseline incidence of major GI bleeding, peptic ulcer and dyspepsia in the general population not receiving aspirin were based on the analysis undertaken for NICE osteoarthritis guideline ^76^ (previously, reported by Copper et al. ^52^).

The RR (1.44 [95%CI, 1.15, 1.81]) of experiencing major GI bleeding events due to ASAVLD was identified from a network meta-analysis^77^. The RR of getting peptic ulcer and dyspepsia was derived from the systematic reviews undertaken for the USPSTF ^78,79^.

|  | Major GI bleeding events | Peptic ulcer | Dyspepsia |
| --- | --- | --- | --- |
| Incidence of harms in general population ^76^ | 0.24% | 0.24% | 26.85% |
| Mean RR of experiencing harms | 1.44 | 1.75 | 1.70 |
| Annual probability of excess harm | 0.0011 | 0.0018 | 0.1880 |

These RRs are assumed to be constant over time while the subject is on ASAVLD. It is probable that the risk of some such events may decrease or increase over time; however, there is very limited evidence to support these assertions.

**S 3.8 Model Parameters for mortality**

**S Table 3.8.1 Age-specific mortality**

Data were extracted from the Global Health Observatory data repository (2016), The World Health Organization, and calculated age-specific mortality rate (ASMR) for Malaysia as per the following method.

| **Expectation of life (LE) at age x-Malaysia** | **LE 2016** | | | **Mortality rate=** | **ASMR** |
| --- | --- | --- | --- | --- | --- |
|  | **Female** | **Male** | **Average LE** | **1/LE** |  |
| 50-54 years | 29.7 | 26.7 | 28.2 | 0.035460993 | 0.03546 |
| 55-59 years | 25.3 | 22.6 | 23.95 | 0.041753653 | 0.04175 |
| 60-64 years | 21.1 | 18.9 | 20 | 0.05 | 0.05000 |
| 65-69 years | 17.1 | 15.3 | 16.2 | 0.061728395 | 0.06173 |
| 70-74 years | 13.3 | 12.1 | 12.7 | 0.078740157 | 0.07874 |
| 75-79 years | 10.1 | 9.4 | 9.75 | 0.102564103 | 0.10256 |
| 80-84 years | 7.2 | 6.9 | 7.05 | 0.141843972 | 0.14184 |
| 85+ years | 5.1 | 4.8 | 4.95 | 0.202020202 | 0.20202 |
| LE, expectation of life; ASMR, age-specific mortality rate. | | | | | |

As per the cause-specific mortality rate in Malaysia, CRC contributes 2.3% of any cause deaths ^80^. Hence, the annual probability of dying from any causes was adjusted to avoid double-counting of CRC deaths by subtracting with 2.3%.

**S Table 3.8.2 Pooled estimates of five-year survival rate (%) of CRC by stage**

| **5-year survival rate by stage (%)** | | | | |
| --- | --- | --- | --- | --- |
| **Author (reference) year (location)** | CRC1 | CRC2 | CRC3 | CRC4 |
| Kong et al. ^81^ 2000-04 (KL) | 78.6 | 52.9 | 44.3 | 9.3 |
| Kong et al. ^81^ 2000-04 (Kuching) | 74.5 | 65 | 36.4 | 5.2 |
| Magaji BA^82^ 2001-2010 (KL)-Malay | 68.68 | 68.68 | 56.18 | 17.98 |
| Magaji BA^82^ 2001-2010 (KL)-Chinese | 63.79 | 63.79 | 45.01 | 13.05 |
| Magaji BA^82^ 2001-2010 (KL)-Indian | 69.95 | 69.95 | 58.85 | NA |
| Hassan MR ^83^ 2008-2010 (Kedah) | 73.4 | 68.3 | 42.6 | 22.1 |
| Ghazali AK ^84^ 1996–2005 (KL) | Unclear | 68.4 | Unclear | NA |
| 2008-2012 (Seremban)- unpublished ^a^ | 67 | 67 | 52 | 22 |
| Pooled estimates of 5-year survival rate | 70.42 (95% CI , 64-79) | 64.55 (95% CI, 53-70) | 48.79 (95% CI, 36-59) | 13.50 (95% CI, 5.2-18) |
| Annual Probability of death due to CRC in Malaysia (SE) | 0.0575 (0.0087) | 0.0684 (0.0099) | 0.0973 (0.0132) | 0.1589 (0.0666) |
| ^a^5-year survival data of patients undergo treatment in Hospital Tuanku Ja'afar, Seremban (2008-2012) . Data extracted from the National Cancer Patient Registry-Colorectal Cancer (NPCR-CC) (shared by authors from an ongoing study at International Medical University, Malaysia).  5 year survival rate for patients with undiagnosed CRC was assumed as lower limit of 95% CI of pooled survival rate.  Abbreviations: CRC, colorectal cancer; KL, Kuala Lumpur; SE, standard error | | | | |

**S 3.9 Utility values**

Evidence on quality of life and corresponding utility scores associated with different stages of CRC from Malaysia was limited. Hence, all utility values were extracted from other appropriate literatures. We have identified two studies that reported health utility scores for patients according to the stage of CRC ^85,86^. The study by Ness et al ^85^ was based on eliciting preferences for hypothetical states from individuals who had previously undergone polypectomy. This study demonstrated a significant difference in utility scores between early and late CRC stages. However, the study by Ramsey et al ^86^ involved eliciting preferences from long-term survivors of CRC. Since guidelines ^87,88^ recommend that utilities should be based on public preferences, utility scores reported by Ness et al^85^ was used in our model. The utility of patients without CRC was obtained from two studies: a cross-sectional study of population-based values for EQ-5D health states in the Malaysian adult population ^89^ and another was a case study among the Malaysian population ^90^. A constant utility score was applied to all non-CRC states.

The utilities were adjusted for harms associated with ASAVLD including peptic ulcer and major gastrointestinal bleed. The impact on quality of life arising from these harms was assumed to have incurred for 1 month within the model ^52^. Relative utilities associated with each of these harms have been derived from the analysis undertaken for the NICE osteoarthritis guidelines ^52,76^. Colonoscopy is known to be associated with certain complications (e.g. perforation) and also required a bowel preparation and recovery period. Hence, a disutility of 0.0025 had been applied to colonoscopy as described previously ^91^.

**S 3.10 Cost data**

The cost analysis comprised only costs directly incurred by the provider only; out-of-pocket expenses and indirect costs due to loss of productivity were excluded from the analysis. Cost estimates used within the analysis have been derived from the amended medical fee schedule 2013 (Private health care facilities and services (amendment) order 2013) ^92^, the consumer price guide (CPG) database, pharmaceutical services program, Ministry of Health, Malaysia ^93^, CRC cost estimates from the University of Malaya Medical Centre (UMMC) and the University Kebangsaan Malaysia Medical Centre (UKMMC)^94–96^, and other relevant literatures. The amended medical fee schedule provides the maximum allowable fees charged by the medical profession for consultations and procedures in Malaysia ^92^. Consumer price guide (CPG) database provides the medicine prices and serves as market price guidance for consumers to make informed choices in Malaysia.

The evidence on resource consumptions and unit costs of CRC services in Malaysia have been previously reported ^94–96^. Based on standard operating procedure (SOP) for CRC according to national data and guidelines, the total provider cost for initial treatment of CRC per year in Malaysian Ringgit (RM) has been previously estimated at the University of Malaya Medical Centre (UMMC, a tertiary hospital in Kuala Lumpur, Malaysia) in 2012 ^94^ as follows: RM 13,485 for stage 1, RM 23,502 for stage 2, RM 25,606 for stage 3, and RM 27,972 for stage 4 per year. The follow-up policies for CRC patients vary considerably across different healthcare settings in Malaysia. Based on the clinical practice guideline (management of colorectal carcinoma) in Malaysia ^97^, our analysis assumed that the patients with CRC would seek outpatient (OP) visits (including monitoring carcinoembryonic antigen (CEA) levels) 3 times per year for the first five years, followed by annual clinic visit. It was also assumed that these patients would repeat colonoscopy at year one and every three to five years thereafter and computed tomography (CT) scan of the thorax, abdomen, and pelvis performed annually for three years. Since the standard unit costs (OP visit, and other investigations) were available from the amended medical fee schedule 2013 ^92^, based on the mean survival (in years) for patients in different stages ^47^, the lifetime costs of CRC was reasonably estimated (Appendix Table 3.10.1). The cost of perforation due to colonoscopy was assumed to be equal to the cost of closure of a perforated ulcer of the duodenum ^92^. The cost of major bleeding was assumed to be equal to the cost of the therapeutic procedure provided by the amended medical fee schedule 2013 ^92^. The costs of treating dyspepsia and peptic ulcer were obtained from a cost analysis conducted at four large tertiary care hospitals in Malaysia ^98^. All costs were converted using the consumer price index (CPI) ^99^ and converted into 2018 Malaysian Ringgit (RM) and then converted in 2018 United States Dollar ($) (conversion rate: 1 US$ = 4.14 RM; date: January 2018; <https://fx-rate.net/USD/MYR/> ).

All costs included in the analysis are presented in Appendix Table 3.10.2.

**S Table 3.10.1 Life-time cost of CRC**

| **Stage** | **Mean survival (year)** | **OP visit (+CEA)** | **COLO** | **CT** | **OP**  **($ 30)** | **COLO**  **($ 383)** | **CT**  **($ 275)** | **Follow-up cost (2018) in US dollar** | **Annual cost of CRC (2018) in US dollar** | **Life time cost (2018) in US dollar** |
| --- | --- | --- | --- | --- | --- | --- | --- | --- | --- | --- |
|  |  | **Average resource use** | | | **Unit cost (2018) x resource used** | | |  |  |  |
| I | 11 | 21 | 4 | 3 | 639 | 1,530 | 824 | 2,993 | 3,701 | 6,695 |
| II | 11 | 21 | 4 | 3 | 639 | 1,530 | 824 | 2,993 | 5,367 | 8,360 |
| III | 8.7 | 18.5 | 3 | 3 | 563 | 1,148 | 824 | 2,535 | 6,785 | 9,320 |
| IV | 1.4 | 4 | 1 | 1.5 | 122 | 383 | 412 | 916 | 7,439 | 8,355 |
| OP=Outpatient; CEA= carcinoembryonic antigen test; COLO=colonoscopy; CT= computed tomography, RM=Ringgit Malaysia  OP visit cost after standardisation (2018)= RM 116 (+CEA cost of RM 10)  Cost of colonoscopy= RM 1435 (RM 1000 + RM 435 for anaesthesia) at 2013, after standardisation at 2018 the cost is RM 1584.  CT (chest, duodenum, and pelvis) with non-ionic contrast = RM 1030, after standardisation at 2018 the cost is RM 1137.  Annual cost of CRC=the cost of treating CRC for the first year after diagnosis after standardisation to 2018.  Life-time cost of CRC excluded new therapies with targeted agents such as monoclonal antibodies, costs for non-incident cases, such as detection and management of recurrences, and ongoing palliative care of stage 4 patients who survive more than a year.  (conversion rate: 1 US$ = 4.14 RM; date: January 2018; <https://fx-rate.net/USD/MYR/> ) | | | | | | | | | | |

**S Table 3.10.2 Cost inputs (in US dollar, year 2018)**

| **Parameter** | **Base case** | **SE** | **Distribution** | **Source** |
| --- | --- | --- | --- | --- |
| Colonoscopy | 383 | 33 | Gamma | The amended medical fee schedule 2013 ^92^ |
| Polypectomy | 109 | 9 | Gamma |  |
| ASAVLD (annual cost) | 35 | 3 | Gamma | The consumer price guide (CPG) database |
| CRC stage I | 6,695 | 580 | Gamma | Azzani M et al. ^94^;  The consumer price guide database (57); appendix table 4.7.1 |
| CRC stage II | 8,360 | 724 | Gamma |  |
| CRC stage III | 9,320 | 807 | Gamma |  |
| CRC stage IV | 8,355 | 723 | Gamma |  |
| Major GI bleeding due to ASAVLD | 347 | 33 | Gamma | The amended medical fee schedule 2013 ^92^ |
| Peptic ulcer due to ASAVLD | 208 | 99 | Gamma | Pok LSL et al. ^98^ |
| Dyspepsia due to ASAVLD | 99 | 8 | Gamma |  |
| Perforation due to colonoscopy | 687 | 59 | Gamma | The amended medical fee schedule 2013 ^92^ |
| Major bleeding due to colonoscopy | 347 | 33 | Gamma |  |
| ASAVLD, aspirin very-low-dose; CRC cost, life-time cost for CRC treatment; GI, gastrointestinal; SE, standard error | | | | |

**S 4.1 Characteristics of studies**

**S Table 4.1.1 Characteristics of RCTs reported advanced adenoma recurrence**

| **Author [Year] (reference)** | **Setting** | **RCT design (double blind placebo controlled)** | **Sample size** | **Age**  **(years)** | **Male%** | **Duration of treatment**  **(follow-up)-years** | **Interventions** | **Outcomes** | | |
| --- | --- | --- | --- | --- | --- | --- | --- | --- | --- | --- |
|  |  |  |  |  |  |  |  | **Advanced adenomas** | **Any adenomas** | **Safety outcome** |
| Baron et al. [1999] ^10^ | US | Parallel (Yes) | 930 | < 80  (mean 61) | 72 | 4 (analysed from end year 1 to end year 4) | CA (1,200 mg/day) | 4/454 | 196/454 | 209/464 |
|  |  |  |  |  |  |  | PLB | 6/459 | 232/459 | 199/466 |
| Bonithon-Kopp et al. [2000] ^100^ | Multi-national | Parallel (Yes) | 439 | 35–75  (mean 59) | 63 | 3 (3) | CA (2,000 mg/day) | 10/176 | 28/176 | 34/204 |
|  |  |  |  |  |  |  | PLB | 8/178 | 36/178 | 21/212 |
| Sandler et al. [2003] ^101^ | US | Parallel (Yes) | 635 | 30-80 | 52 | ≈3 years (3 to 4 years) | ASALD | 7/259 | 43/259 | 25/317 |
|  |  |  |  |  |  |  | PLB | 9/258 | 70/258 | 24/318 |
| Arber et al. [2006] ^102^ | Multi-national | Parallel (Yes) | 1,561 | 30–92  (mean 61) | 66 | ≈3 years (1 and 3 years after the baseline examination) | Cele400 | 42/840 | 270/840 | 186/933 |
|  |  |  |  |  |  |  | PLB | 56/557 | 264/557 | 106/628 |
| Bertagnolli et al. [2006] ^103^ | Multi-national | Parallel three arms (Yes) | 635 | 30-80 | 52 | ≈3 years (1 and 3 years after the baseline examination) | Cele400 | 44/613 | 44/613 | 139/683 |
|  |  |  |  |  |  |  | Cele800 | 35/601 | 35/601 | 154/669 |
|  |  |  |  |  |  |  | PLB | 99/608 | 99/608 | 127/676 |
| Baron et al. [2003] /  Cole et al. [2007] ^27,28^ | US | Three by-  two factorial (Yes)^a^ | 1,021 | 21-81  (mean 57) | 64 | 3 (3) | ASALD | 37/323 | 147/323 | 32/201 |
|  |  |  |  |  |  |  | ASAVLD | 21/334 | 123/334 | 27/203 |
|  |  |  |  |  |  |  | CTL | 41/330 | 157/330 | 28/202 |
| Logan et al. [2008] ^104^ | UK,  Denmark | Two by-  two factorial (Yes) ^b^ | 939 | 28–75  (mean 58) | 56 | 3 (3) | ASALD | 41/434 | 99/434 | 130/472 |
|  |  |  |  |  |  |  | CTL | 63/419 | 121/419 | 123/467 |
| Chu et al. [2011] ^105^ | US | Parallel (Yes) | 220 | > 18  (median 68) | 63 | 5 (5) | CA (CaCO3 1800 mg/day) | 17/95 | 42/95 | 4/95 |
|  |  |  |  |  |  |  | PLB | 19/99 | 62/99 | 1/99 |
| Benamouzig et al. [2012] ^12^ | France | Parallel (Yes) | 272 | 18-75 | 70 | 4 (4) | ASALD | 18/128 | 65/128 | 15/140 |
|  |  |  |  |  |  |  | PLB | 18/116 | 62/116 | 17/132 |
| Ishikawa et al. [2014] ^74^ | Japan | Parallel (Yes) | 389 | 40-70 | 79 | 2 (2) | ASAVLD | 3/152 | 56/152 | 0/191 |
|  |  |  |  |  |  |  | PLB | 4/159 | 73/159 | 0/198 |
| Baron et al. [2015] ^106^ | US | Two by-  two factorial (Yes) | 2,259 | 45–75  (mean 59) | 85 | 5 (at year 3or 5) | CA (1,200 mg/day) | 63/662 | 259/662 | 272/714 |
|  |  |  |  |  |  |  | VD | 42/384 | 179/384 | 134/420 |
|  |  |  |  |  |  |  | CA+VD | 56/648 | 259/648 | 229/710 |
|  |  |  |  |  |  |  | PLB | 35/380 | 183/380 | 119/415 |
| P. A. Thompson et al. [2016] ^107^ | US | Two by-  two factorial (Yes) | 824 | Mean 63 | 68 | 1 (Within 1 year of discontinuing intervention)^d^ | Cele400 | 3/119 | 34/123 | 53/401 |
|  |  |  |  |  |  |  | PLB | 12/119 | 50/121 | 41/397 |
| Pommergaard et al. [2016] ^108^ | Multi-national | Parallel (Yes) | 427 | Mean 59 | 58 | 3 (3) | ASAVLD+ CA+VD | 5/209 | 52/209 | 60/352 |
|  |  |  |  |  |  |  | PLB | 7/218 | 58/218 | 67/362 |
| Hull et al. [2018] ^109^ | UK | Two by-  two factorial (Yes) | 709 | 62-69  (mean 65) | 80 | 1 (after 1 year) | ASALD | 18/324 | 198/324 | 17/344 |
|  |  |  |  |  |  |  | PLB | 19/316 | 197/316 | 25/353 |
| Abbreviations: ASALD, aspirin low-dose; ASAVLD, aspirin very low-dose; CA, calcium; Cele400, celecoxib 400 mg/day; Cele800, celecoxib 800 mg/day; CTL, control; PLB, placebo; US, The United states of America; UK, the United Kingdom; VD, vitamin D. | | | | | | | | | | |

**S Table 4.1.2 other characteristics of included studies**

| **Author [Year]** | **Population** | **Definition of advanced adenomas** | **% with advanced neoplasia at**  **baseline** | **Risk of bias** |
| --- | --- | --- | --- | --- |
| Baron et al. [1999] ^10^ | History of adenomas; and all polyps removed and pathologically examined within three months | Not defined; identified based on severe atypia or cancer | NA | Low |
| Bonithon-Kopp et al. [2000] ^100^ | History of adenomas; and documented clean colon post-polypectomy | Villous or tubule-villous features , or an estimated diameter of at least 1cm | 56-59% | Low |
| Sandler et al. [2003] ^101^ | Histologically documented colon or rectal cancer with a low risk of recurrent disease; and documented clean colon post-polypectomy | Adenomas at least 1 cm in diameter or had villous components | 63% with Dukes  A or B1 CRC | Low |
| Arber et al. [2006] ^102^ | History of adenomas; and documented clean colon post-polypectomy within three months | Adenoma >1 cm (villous or tubule-villous histology); high-grade dysplasia; intra-mucosal carcinoma or invasive cancer | 48% | Low |
| Bertagnolli et al. [2006] ^103^ | History of either multiple adenomas or removal of a single adenoma ≥ 0.5 cm in diameter; and documented clean colon post-polypectomy within six months | Adenoma >1 cm (villous or tubule-villous histology); high-grade dysplasia; Intra-mucosal carcinoma or invasive cancer | 43% | Low |
| Baron et al. [2003] /  Cole et al. [2007] ^27,28^ | History of adenomas; and documented clean colon post-polypectomy within three months | Tubule-villous adenomas (25-75% villous), villous adenomas (> 75% villous), adenomas ≥ 1 cm in diameter, severe dysplasia, or invasive cancer | 28-29% | Low |
| Logan et al. [2008] ^104^ | History of colorectal adenoma >0.5 cm; and documented clean colon post-polypectomy. | Adenomas that were either 1 cm or larger in diameter, villous or tubule-villous, or showed severe dysplasia or invasive cancer | 68% | Low |
| Chu et al. [2011] ^105^ | History of stage 0, I or II CRC and complete resection of CRC within 550 days and a clearing colonoscopy within 180 days | Villous or tubule-villous features , or an estimated diameter of at least 1cm | 39-46% with  stage-2 CRC | High |
| Benamouzig et al. [2012] ^12^ | History of at least three adenomas irrespective of size, or at least one measuring 6 mm in diameter or more; and documented clean colon post-polypectomy within three months | Adenomas with a maximum diameter of at least 1 cm, at least 25% villous elements or evidence of high-grade dysplasia | 69% | Unclear |
| Ishikawa et al. [2014] ^74^ | History of single/multiple colorectal adenomas and/or adenocarcinomas with invasions confined to the mucosa; and documented clean colon post-polypectomy within three months | Adenomas with high grade dysplasia | 26% with CRC | Low |
| Baron et al. [2015] ^106^ | History of adenomas; and documented clean colon post-polypectomy within six months | Adenomas with cancer, high-grade dysplasia, more than 25% villous features, or an estimated diameter of at least 1cm | 19% | Low |
| P. A. Thompson et al. [2016] ^107^ | History of one or more colorectal adenomas 0.3 cm or larger; and documented clean colon post-polypectomy within six months | 1 cm or larger, with tubulovillous or villous tissue architecture, and/or with high-grade dysplasia | 32% | High |
| Pommergaard et al. [2016] ^108^ | History of one or more sporadic adenomas removed from the colon or rectum within the last 3 months | ≥ 1 cm diameter or high grade dysplasia | 16% with high grade dysplasia | Low |
| Hull et al. [2018] ^109^ | History of colorectal adenomas; and documented clean colon post-polypectomy | ≥ 1 cm diameter, high-grade dysplasia, or  villous histology | NA | Low |

**S 4.2 Risk of bias assessment of RCTs using revised Cochrane ROB assessment tool**

| **Author, year** | **A** | **B** | **C** | **D** | **E** | **F** |
| --- | --- | --- | --- | --- | --- | --- |
| Baron et al. (1999) | + | + | + | + | + | **+** |
| Bonithon-Kopp et al. (2000) | + | + | + | + | + | **+** |
| Sandler et al. (2003) | + | + | + | + | + | **+** |
| Arber et al. (2006) | + | + | + | + | + | **+** |
| Bertagnolli et al. (2006) | + | + | + | + | + | **+** |
| Cole et al. (2007) | + | + | + | + | + | **+** |
| Logan et al. (2008) | + | + | + | + | + | **+** |
| Chu et al. (2011) | - | + | + | ? | + | **-** |
| Benamouzig et al. (2012) | + | + | ? | + | + | **?** |
| Ishikawa et al. (2014) | + | + | + | + | + | **+** |
| Baron et al. (2015) | + | + | + | + | + | **+** |
| P. A. Thompson et al. (2016) | + | ? | - | + | + | **+** |
| Pommergaard et al. (2016) | + | + | + | + | + | **+** |
| Hull et al. (2018) | + | + | + | + | + | **+** |
| A-Bias arising from the randomizsation randomisation process; B- Bias due to deviations from intended interventions; C- Bias due to missing outcome data; D- Bias in measurement of the outcome; E-Bias in selection of the reported result; F-Overall bias. | | | | | | |
| **+symbol/green colour means ‘low risk of bias’; symbol/yellow colour means ‘some concerns’; - symbol/red colour means ‘high risk of bias’.** | | | | | | |

**S 4.3 Results from pairwise meta-analyses of efficacy and safety outcomes**

**S Figure 4.3.1 Pairwise meta-analysis: recurrence of advanced adenomas**

**S Figure 4.3.2 Pairwise meta-analysis: serious adverse events**

**S 4.4 Network meta-analysis of CPAs: Efficacy outcomes**

**S Table 4.4.1 Network meta-analysis: recurrence of advanced adenomas**

| **Intervention** | **Recurrence of advanced adenomas** | |
| --- | --- | --- |
|  | **RR (95% CI)** | **SUCRA rank** |
| **Cele800** | **0.36 (0.25,0.52)** | 1 |
| **Cele400** | **0.45 (0.35,0.58)** | 2 |
| **ASAVLD** | **0.49 (0.31,0.78)** | 3 |
| ASALD | 0.79 (0.63,1.00) | 4 |
| ASAVLD+CA+VD | 0.75 (0.24,2.31) | 5 |
| CA+VD | 0.92 (0.64,1.33) | 6 |
| CA | 1.00 (0.74,1.35) | 7 |
| PLB | Reference | 8 |
| VD | 1.17 (0.79,1.73) | 9 |
| Overall inconsistency chi square (P value) | 1.28 (0.86) | |
| Number of studies | 14 | |
| RR (95%CI) <1 indicates that the treatment is effective.  Abbreviations: ASA-VLD, very-low-dose-aspirin; ASA-LD, low-dose-aspirin; Ca, calcium; Cele, celecoxib (400 mg and 800 mg daily), PLB, placebo; VD, vitamin D. | | |

**S Figure 4.4.1 SUCRA ranking curve: recurrent advanced adenomas**


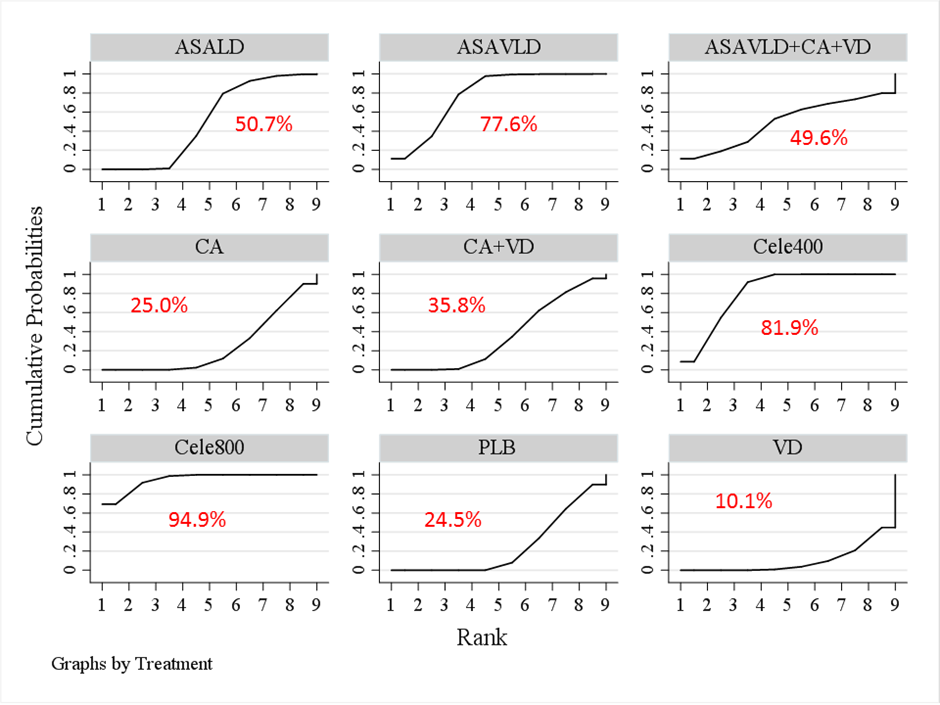


Abbreviations: ASA-VLD, very-low-dose-aspirin; ASA-LD, low-dose-aspirin; Ca, calcium; Cele, celecoxib (400 mg and 800 mg daily), PLB, placebo; VD, vitamin D. Higher SUCRA scores (in %) correspond to a higher ranking for prevention of recurrent colorectal advanced adenomas.

**S Table 4.4.2 Sensitivity analyses of primary efficacy outcome**

| **Intervention** | **Primary analysis: all trials** | | **Using Low risk of bias trials** | | **Excluding population with h/o of CRC** | | **Excluding trials with follow-up <2years** | |
| --- | --- | --- | --- | --- | --- | --- | --- | --- |
|  | **RR (95%CI)** | **Rank** | **RR (95%CI)** | **Rank** | **RR (95%CI)** | **Rank** | **RR (95%CI)** | **Rank** |
| **Cele800** | 0.36 (0.25,0.52) | 1 | 0.36 (0.25,0.52) | 1 | 0.36 (0.25,0.52) | 1 | 0.36 (0.25,0.52) | 1 |
| **Cele400** | 0.45 (0.35,0.58) | 2 | 0.45 (0.35,0.58) | 2 | 0.45 (0.35,0.58) | 2 | 0.45 (0.35,0.58) | 2 |
| **ASAVLD** | 0.49 (0.31,0.78) | 3 | 0.49 (0.31,0.78) | 3 | 0.49 (0.31,0.78) | 3 | 0.49 (0.31,0.77) | 3 |
| **ASALD** | 0.79 (0.63,1.00) | 4 | 0.79 (0.63,1.00) | 4 | 0.79 (0.63,1.00) | 4 | 0.77 (0.61,0.98) | 4 |
| **ASAVLD+**  **CA+VD** | 0.75 (0.24,2.31) | 5 | 0.75 (0.24,2.31) | 5 | 0.75 (0.24,2.31) | 5 | 0.75 (0.24,2.31) | 5 |
| **CA+VD** | 0.92 (0.64,1.33) | 6 | 0.94 (0.64,1.38) | 6 | 0.94 (0.64,1.38) | 6 | 0.92 (0.64,1.33) | 6 |
| **CA** | 1.00 (0.74,1.35) | 7 | 1.03 (0.73,1.46) | 7 | 1.03 (0.73,1.46) | 7 | 1.00 (0.74,1.35) | 7 |
| **PLB** | Reference | 8 | Reference | 8 | Reference | 8 | Reference | 8 |
| **VD** | 1.17 (0.79,1.73) | 9 | 1.19 (0.79,1.78) | 9 | 1.19 (0.79,1.78) | 9 | 1.19 (0.79,1.78) | 9 |
| When trials with follow-up less than 2 years excluded from NMA, ASALD become statistically significant. | | | | | | | | |

**S Figure 4.4.2 Pairwise (upper right portion) and network (lower left portion) meta-analytic results for advanced adenoma recurrence**

| ASALD | **1.81**  **(1.09,3.02)** | NA | NA | NA | NA | NA | NA | **0.79 (0.63,0.99)** |
| --- | --- | --- | --- | --- | --- | --- | --- | --- |
| **1.61 (1.01,2.55)** | ASAVLD | NA | NA | NA | NA | NA | NA | **0.53**  **(0.33,0.85)** |
| 1.06 (0.34,3.37) | 0.66 (0.20,2.24) | ASAVLD+CA+ VD | NA | NA | NA | NA | NA | 0.75  (0.24,2.31) |
| 0.79 (0.54,1.15) | **0.49 (0.29,0.85)** | 0.74 (0.23,2.39) | CA | 1.10  (0.78,1.55) | NA | NA | 0.87  (0.60,1.26) | 1.00  (0.74,1.35) |
| 0.86 (0.56,1.33) | **0.54 (0.30,0.96)** | 0.81 (0.25,2.66) | 1.09 (0.78,1.52) | CA+VD | NA | NA | NA | 0.94  (0.63,1.40) |
| **1.75 (1.25,2.45)** | 1.09 (0.65,1.82) | 1.64 (0.52,5.24) | **2.22 (1.50,3.27)** | **2.03 (1.30,3.17)** | Cele400 | 1.23  (0.80,1.89) | NA | **0.45**  **(0.35,0.58)** |
| **2.20 (1.43,3.37)** | 1.37 (0.77,2.44) | 2.07 (0.63,6.78) | **2.79 (1.74,4.46)** | **2.56 (1.52,4.28)** | 1.26 (0.84,1.87) | Cele800 | NA | **0.36**  **(0.25,0.52)** |
| 0.68 (0.43,1.07) | **0.42 (0.23,0.77)** | 0.64 (0.19,2.12) | 0.86 (0.60,1.23) | 0.79 (0.54,1.15) | **0.39 (0.24,0.62)** | **0.31 (0.18,0.53)** | VD | 1.19  (0.78,1.82) |
| **0.79 (0.63,1.00)** | **0.49 (0.31,0.78)** | 0.75 (0.24,2.31) | 1.00 (0.74,1.35) | 0.92 (0.64,1.33) | **0.45 (0.35,0.58)** | **0.36 (0.25,0.52)** | 1.17 (0.79,1.73) | PLB |

Outcomes are expressed as risk ratios (95% confidence intervals). For the pairwise meta-analyses, risk ratio less than 1 indicate that the treatment specified in the row is more efficacious. For the network meta-analysis, risk ratio less than 1 indicate that the treatment specified in the column is more efficacious. Bold green results indicate statistical significance.

**S Figure 4.4.3 Predictive interval plot**

We have calculated the 95% prediction intervals taking into account the heterogeneity and the range in which the underlying true effect size of future trials would lie with 95% certainty^110^. Inference: the results for ASAVLD and celecoxib were consistent.

**S 4.5 Network meta-analysis of CPAs: Serious adverse events**

**S Table 4.5.1 Network meta-analysis of CPAs: Serious adverse events**

| **Intervention** | **Serious adverse events (SAEs)** | |
| --- | --- | --- |
|  | **RR (95% CI)** | **SUCRA rank** |
| ASAVLD | 0.82 (0.47,1.41) | 1 |
| ASAVLD+CA+VD | 0.92 (0.64,1.32) | 2 |
| ASALD | 0.97 (0.78,1.21) | 3 |
| PLB | Reference | 4 |
| VD | 1.05 (0.82,1.35) | 5 |
| CA+VD | 1.07 (0.85,1.34) | 6 |
| Cele400 | 1.16 (0.97,1.38) | 7 |
| CA | **1.22 (1.02,1.44)** | 8 |
| Cele800 | **1.27 (1.00,1.62)** | 9 |
| Overall inconsistency chi square (P value) | 1.04 (0.79) | |
| Number of studies | 14 | |

**S Figure 4.5.1 SUCRA ranking curve: Serious adverse events**


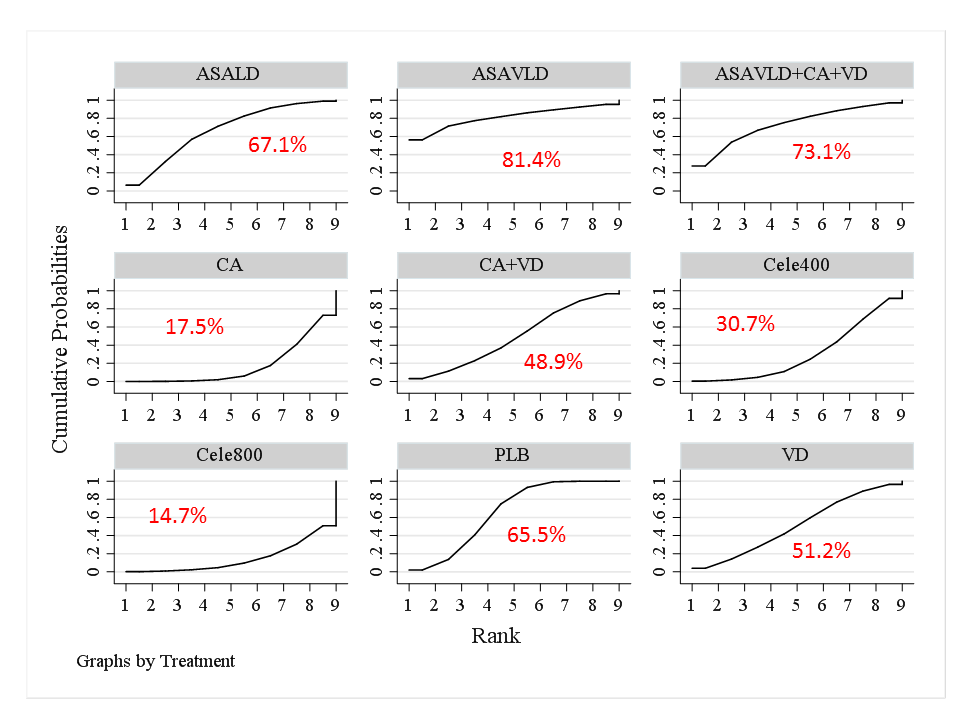


Abbreviations: ASA-VLD, very-low-dose-aspirin; ASA-LD, low-dose-aspirin; Ca, calcium; Cele, celecoxib (400 mg and 800 mg daily), PLB, placebo; VD, vitamin D. Higher SUCRA scores (in %) correspond to a higher ranking for safety.

**S Table 4.5.2 Network meta-analysis of CPAs: Cardiovascular (CV) events**

| **Intervention** | **Outcome: CV events** | | |
| --- | --- | --- | --- |
|  | **RR [95% CI]** | **SUCRA score** | **Ranking** |
| Cele800 | 2.63 (1.28,5.33) | 0.10 | 6 |
| Cele400 | 1.70 (0.96,2.99) | 0.32 | 5 |
| ASAVLD | 0.98 (0.37,2.60) | 0.73 | 2 |
| ASALD | 1.86 (0.96,3.64) | 0.28 | 4 |
| CA | 1.09 (0.47,2.56) | 0.66 | 3 |
| PLB | Reference | 0.78 | 1 |
| Overall inconsistency  Chi-square (p value) | 2.91 (0.41) | | |
| Abbreviations: ASA-VLD, very-low-dose-aspirin; ASA-LD, low-dose-aspirin; Ca, calcium; Cele, celecoxib (400 mg and 800 mg daily), PLB, placebo; VD, vitamin D. | | | |

**S 4.6 Trial sequential analysis (TSA)**

Trial sequential analyses (TSAs) were performed for those CPAs which have demonstrated evidence of efficacy for advanced adenomas in the pairwise meta-analyses based on an assumption of 9% of control group event proportion (incidence of recurrent advanced adenomas in the control group from all trials) with type 1 error of 5% (two-sided) and type II error of 20%.

**S Figure 4.6.1 TSA on ASAVLD versus placebo on advanced adenoma recurrence**

**
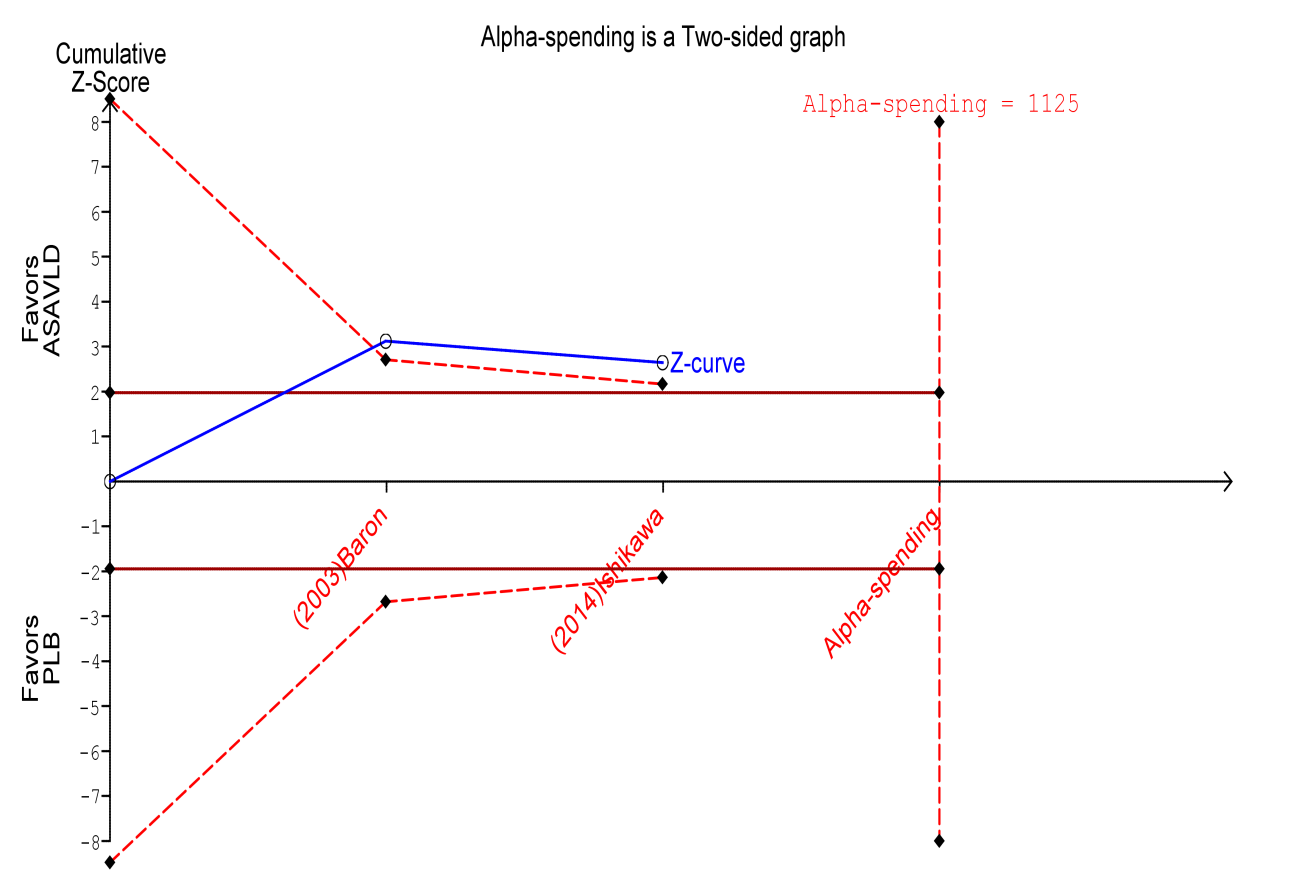
**

The TSA is based on the pairwise meta-analysis of ASAVLD versus placebo (based on two RCTs) as shown in Appendix Figure 4.3.1. In a meta-analysis, where the risk of random error is of specific concern at initial stages, the O’Brien-Fleming α-spending boundaries (trial sequential monitoring boundaries) have been used in TSA for testing statistical significance before the information size has been reached ^41,42^.

The sample size required to demonstrate a 47% relative reduction was 1,125 participants. The number of participants included in the pairwise meta-analysis from two trials (n=975) did not exceed the required sample size. However, the Z-curve (blue line) had crossed the trial sequential monitoring boundary (red dotted line). Hence the evidence obtained from the meta-analysis is considered conclusive.

**S Figure 4.6.2 TSA on ASALD versus placebo on advanced adenoma recurrence**


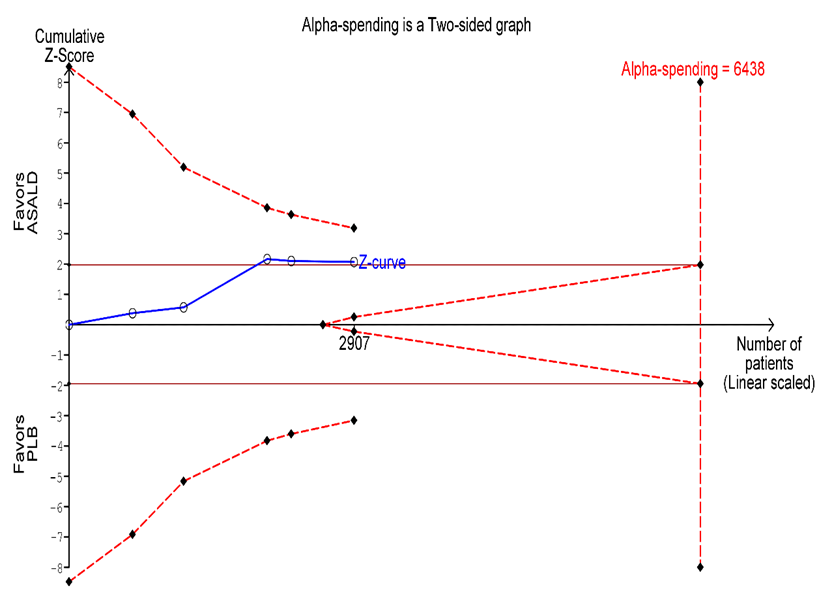


For ASALD, the sample size required to demonstrate a 21% relative reduction (obtained from the pairwise meta-analysis; Appendix Figure 4.3.1) based on the aforementioned assumptions was 6,438 subjects. Although, the Z-curve surpassed the conventional boundary with a cumulative Z-statistic above 1.96, the number of participants included in the meta-analysis (n=2,907) did not exceed the required sample size (that is, 6,438) or did not cross the alpha-spending monitoring boundary. Hence the cumulative evidence for ASALD from the pairwise meta-analysis is considered inconclusive. More trials with ASALD are required to confirm the effect.

**S Figure 4.6.3 TSA on Celecoxib versus placebo on advanced adenoma recurrence**

Trial sequential boundary was not formed. The sample size required to demonstrate a 65% relative reduction was achieved from one trial (Arber 2008; Appendix Figure 4.3.1). There is no further evaluation required. Evidence is conclusive.

**S** **4.7 Network consistency and small study effects**

**S Table 4.7.1 Network consistency**

| **Assessment of global inconsistency in networks using the ‘design-by-treatment’ interaction model.** | | |
| --- | --- | --- |
| **Network outcome** | **Chi-square** | **P value for test of global inconsistency** |
| Recurrence of advanced adenomas | 1.28 | 0.86 |
| Serious adverse events | 1.04 | 0.79 |
| Cardiovascular events | 2.91 | 0.41 |
| P>0.05 indicates there is no statistically significant inconsistency among trials included in NMAs. | | |

**S Figure 4.7.1 Comparison-adjusted plot for small study effects: primary outcome**

**S Figure 4.7.1 Comparison-adjusted plot for small study effects: safety outcome**

**S 4.8 GRADE summary of evidence for primary outcome**

| **Comparisons** | **Direct evidence**  **(from pairwise meta-analysis)** | | **Indirect evidence**  **(from 1^st^ order loops)** | | **Difference** | **Network meta-analysis** | |
| --- | --- | --- | --- | --- | --- | --- | --- |
| **Recurrence of advanced adenomas** | **RR**  **[95% CI]** | **Quality of evidence** | **RR**  **[95% CI]** | **Quality of evidence** | **P value** | **RR**  **[95% CI]** | **Quality of evidence** |
| ASA-LD vs. Placebo | 0.79 (0.63,0.99) | Low ^b, e^ | 0.46 (0.02, 10.13) | Low^c^ | 0.51 | 0.79 (0.63,1.00) | Low |
| ASA-VLD vs. Placebo | 0.53 (0.33,0.85) | Moderate^e^ | 0.33 (0.13, 0.86) | Low^c^ | 0.35 | 0.49 (0.31,0.78) | Moderate |
| Celecoxib (400 mg/day) vs Placebo | 0.45 (0.35,0.58) | High | NA | NA | NA | 0.45 (0.35,0.58) | High |
| Celecoxib (800mg/day) vs Placebo | 0.36 (0.25,0.52) | High | 0.40 (0.14, 0.91) | NA | 0.814 | 0.36 (0.25,0.52) | High |
| ASA-LD vs ASAVLD | 1.27 (1.09,3.03) | Low  ^b, e^ | 1.11 (0.48, 2.54) | Low^c^ | 0.29 | 1.61 (1.01,2.55) | Low |
| Randomised controlled trials (RCTs) without important limitations are rated high on the GRADE scale. Further rating is based on following criteria:   1. Risk of bias 2. Imprecision 3. Based on rating of the two pairwise estimates that contributes to the indirect estimate (first order loop) 4. Intransitivity 5. Indirectness 6. Co-intervention effect 7. Inconstancy 8. Publication bias | | | | | | | |

**S 4.9 Absolute anticipated benefits and risks of CPAs**

| **Interventions with evidence of efficacy ^a^** | **Anticipated absolute risk difference of advanced adenomas (95% CI) in individuals at different risk group at baseline** | | **Anticipated absolute risk difference of adverse events per 1000 individuals (95% CI)** |
| --- | --- | --- | --- |
|  | **Non-advanced adenoma at baseline (low-risk group) ^b^** | **Advanced adenoma at baseline (high-risk group) ^c^** | **Serious adverse events ^d^** |
| **Celecoxib 400 mg/day** | -41 (-31 to -48) | -90 (-68 to -106) | 21 (0 to 46) |
| **Celecoxib 800 mg/day** | -47 (-36 to -56) | -104(-78 to -122) | 32 (1 to 71) |
| **ASAVLD** | -35(-11 to -51) | -77 (-24 to -109) | -6 (-57 to 78) |
| **ASALD** | -16 (-1 to -27) | -34 (-2 to -60) | 1 (21 to 27) |
| ^a^ Listed interventions all demonstrated statistically significant reduction in recurrence of advanced adenomas as per Table 3.3. ASALD was also included in this analysis due to its marginal significance; ^b^ low risk group includes patients with 1-2 small (<1 cm) tubular adenoma(s) with low grade dysplasia, and estimated risk of advanced adenomas of 74 per 1000 individuals without intervention; ^c^ high risk group includes patients with 1 cm or larger, with villous or tubule-villous histology, with high grade dysplasia, and/or with intra-mucosal carcinoma or invasive cancer, and estimated risk of advanced adenomas of 163 per 1000 individuals without intervention; ^d^ serious adverse events were defined as events resulting in death, hospital admission because of an adverse event, severe gastrointestinal bleeding, CV or non-CV complications, or discontinuation of intervention due to an adverse event or events that were defined as serious or severe by study authors. 190 per 1000 events graded as serious or severe by original study authors over same time period without any intervention.  Abbreviations: ASA-VLD, very-low-dose-aspirin; ASA-LD, low-dose-aspirin  This analysis was conducted based on the method previously described by Dulai S et al. ^43^ | | | |

**S 4.10 Scatter plot for SUCRA rankings for primary efficacy and safety outcomes**

**
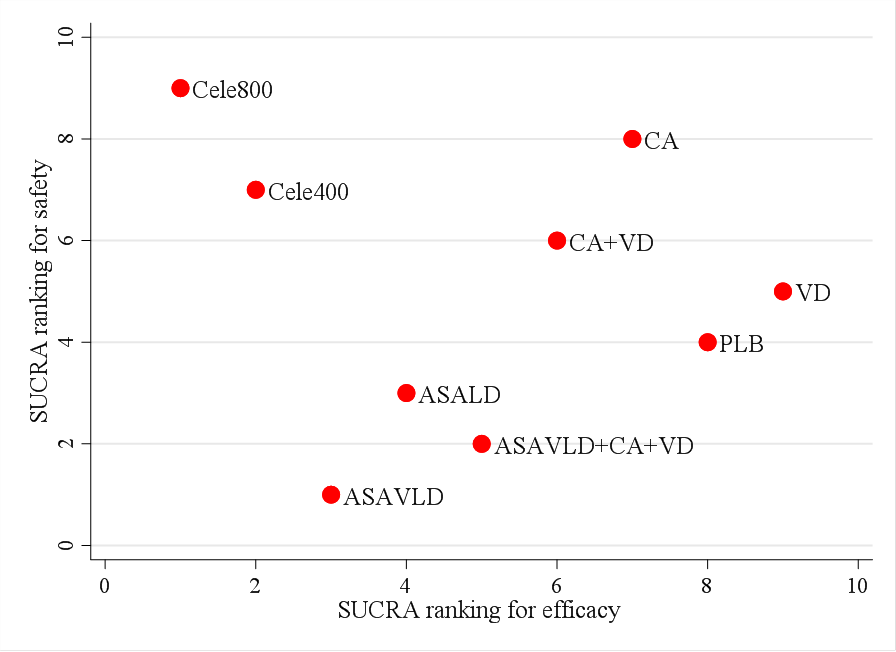
**

Treatments lying in the lower right corner are more effective and acceptable than the other treatments.

Abbreviations: ASA-LD, low-dose-aspirin; ASA-VLD, very-low-dose-aspirin; Ca, calcium; Cele, celecoxib (400 mg and 800 mg daily), PLB, placebo; SUCRA, surface under the cumulative ranking curve; VD, vitamin D

**S 5 Cost-effectiveness analysis**

**S Table 5.1 Occurrence of CRC cases over lifetime for different strategies: Base case analysis**

| **Cases of CRC/100,000 over lifetime (Compliance to colonoscopy: 60%)** | | | | | | | | | | | |
| --- | --- | --- | --- | --- | --- | --- | --- | --- | --- | --- | --- |
| **Year** | **No screening** | | | **ASAVLD** | | **Surveillance Colonoscopy** | | | **ASAVLD+**  **colonoscopy** | | |
|  | **New CRC** | **Late CRC** | | **New CRC** | **Late CRC** | **New CRC** | | **Late CRC** | **New CRC** | | **Late CRC** |
| 0 | 0 | 0 | | 0 | 0 | 0 | | 0 | 0 | | 0 |
| 1 | 0 | 0 | | 0 | 0 | 0 | | 0 | 0 | | 0 |
| 2 | 0 | 0 | | 0 | 0 | 0 | | 0 | 0 | | 0 |
| 3 | 0 | 0 | | 0 | 0 | 0 | | 0 | 0 | | 0 |
| 4 | 0 | 0 | | 0 | 0 | 0 | | 0 | 0 | | 0 |
| 5 | 5 | 0 | | 3 | 0 | 2 | | 0 | 1 | | 0 |
| 6 | 17 | 0 | | 10 | 0 | 9 | | 0 | 3 | | 0 |
| 7 | 34 | 1 | | 20 | 1 | 186 | | 0 | 53 | | 0 |
| 8 | 55 | 4 | | 33 | 2 | 17 | | 1 | 6 | | 0 |
| 9 | 78 | 8 | | 47 | 5 | 28 | | 2 | 8 | | 1 |
| 10 | 102 | 15 | | 63 | 9 | 367 | | 2 | 101 | | 1 |
| 11 | 124 | 22 | | 79 | 13 | 26 | | 3 | 8 | | 1 |
| 12 | 158 | 31 | | 104 | 19 | 41 | | 5 | 11 | | 1 |
| 13 | 186 | 39 | | 125 | 25 | 494 | | 3 | 135 | | 1 |
| 14 | 207 | 50 | | 143 | 32 | 33 | | 5 | 10 | | 1 |
| 15 | 225 | 61 | | 159 | 40 | 45 | | 7 | 13 | | 2 |
| 16 | 236 | 70 | | 171 | 48 | 501 | | 4 | 140 | | 1 |
| 17 | 279 | 78 | | 209 | 54 | 38 | | 6 | 12 | | 2 |
| 18 | 303 | 84 | | 233 | 60 | 54 | | 8 | 15 | | 2 |
| 19 | 316 | 95 | | 248 | 69 | 607 | | 5 | 174 | | 1 |
| 20 | 320 | 105 | | 257 | 79 | 39 | | 7 | 12 | | 2 |
| 21 | 314 | 111 | | 258 | 85 | 51 | | 9 | 15 | | 3 |
| 22 | 317 | 114 | | 267 | 89 | 582 | | 5 | 170 | | 1 |
| 23 | 312 | 114 | | 268 | 92 | 37 | | 6 | 12 | | 2 |
| 24 | 302 | 115 | | 265 | 94 | 49 | | 8 | 15 | | 2 |
| 25 | 289 | 114 | | 259 | 96 | 530 | | 5 | 158 | | 1 |
| 26 | 269 | 110 | | 246 | 94 | 33 | | 6 | 11 | | 2 |
| 27 | 261 | 105 | | 245 | 92 | 44 | | 7 | 14 | | 2 |
| 28 | 247 | 98 | | 237 | 88 | 56 | | 9 | 17 | | 3 |
| 29 | 230 | 94 | | 225 | 86 | 69 | | 12 | 23 | | 4 |
| 30 | 212 | 89 | | 212 | 83 | 82 | | 16 | 31 | | 5 |
| 31 | 186 | 80 | | 189 | 77 | 90 | | 19 | 39 | | 6 |
| 32 | 164 | 71 | | 170 | 70 | 97 | | 22 | 47 | | 8 |
| 33 | 144 | 63 | | 152 | 63 | 101 | | 24 | 55 | | 10 |
| 34 | 125 | 56 | | 136 | 56 | 102 | | 27 | 61 | | 12 |
| 35 | 109 | 49 | | 121 | 51 | 101 | | 28 | 65 | | 14 |
| 36 | 82 | 37 | | 93 | 39 | 86 | | 26 | 59 | | 14 |
| 37 | 62 | 28 | | 72 | 31 | 72 | | 23 | 53 | | 13 |
| 38 | 47 | 21 | | 57 | 24 | 60 | | 20 | 47 | | 13 |
| 39 | 36 | 16 | | 44 | 18 | 49 | | 17 | 41 | | 11 |
| 40 | 27 | 12 | | 35 | 14 | 40 | | 14 | 36 | | 10 |
| 41 | 21 | 9 | | 27 | 11 | 32 | | 12 | 31 | | 9 |
| 42 | 16 | 7 | | 21 | 9 | 26 | | 10 | 26 | | 8 |
| 43 | 12 | 5 | | 17 | 7 | 21 | | 8 | 22 | | 7 |
| 44 | 9 | 4 | | 13 | 5 | 16 | | 6 | 18 | | 6 |
| 45 | 7 | 3 | | 10 | 4 | 13 | | 5 | 15 | | 5 |
| 46 | 5 | 2 | | 8 | 3 | 10 | | 4 | 12 | | 4 |
| 47 | 4 | 2 | | 6 | 3 | 8 | | 3 | 10 | | 4 |
| 48 | 3 | 1 | | 5 | 2 | 6 | | 3 | 8 | | 3 |
| 49 | 2 | 1 | | 4 | 2 | 5 | | 2 | 7 | | 2 |
| 50 | 2 | 1 | | 3 | 1 | 4 | | 2 | 5 | | 2 |
| 51 | 1 | 0 | | 1 | 1 | 2 | | 1 | 3 | | 1 |
| 52 | 0 | 0 | | 1 | 0 | 1 | | 0 | 1 | | 0 |
| 53 | 0 | 0 | | 0 | 0 | 0 | | 0 | 1 | | 0 |
| 54 | 0 | 0 | | 0 | 0 | 0 | | 1 | 0 | | 0 |
| 55 | 0 | 0 | | 0 | 0 | 0 | | 0 | 0 | | 1 |
| 56 | 0 | 0 | | 0 | 0 | 0 | | 0 | 0 | | 0 |
| 57 | 0 | 0 | | 0 | 0 | 0 | | 0 | 0 | | 0 |
| 58 | 0 | 0 | | 0 | 0 | 0 | | 0 | 0 | | 0 |
| 59 | 0 | 0 | | 0 | 0 | 0 | | 0 | 0 | | 0 |
| 60 | 0 | 0 | | 0 | 0 | 0 | | 0 | 0 | | 0 |
|  | **6462** | **2201** | | **5572** | **1843** | **4962** | | **413** | **1832** | | **204** |
| **CRC case prevented** | | | | **890** | **358** | **1500** | | **1788** | **4630** | | **1997** |
| **CRC prevention rate (%) compared with no screening** | | | | **14%** | **16%** | **23%** | | **81%** | **72%** | | **91%** |
| **New cases of CRC prevention rate (%): Combination strategy vs COLO alone** | | | | | | **4962-1832= 3130 cases prevented** | | | | | **63%** |
| Late CRC case: stage IV CRC; new CRC case: CRC at any stage | | | | | | | | | | | |
| **Compliance to colonoscopy: 100%**  **New cases of CRC prevention rate (%)** | | | **ASAVLD** | | | | **Surveillance Colonoscopy** | | | **ASAVLD+**  **colonoscopy** | |
|  |  |  | **14%** | | | | **56.8%** | | | **80%** | |

**S Figure 5.1 Tornado diagram illustrating the one-way sensitivity analysis results of combination strategy (ASAVLD with surveillance colonoscopy) compared to no screening for incremental cost (in US dollar)**


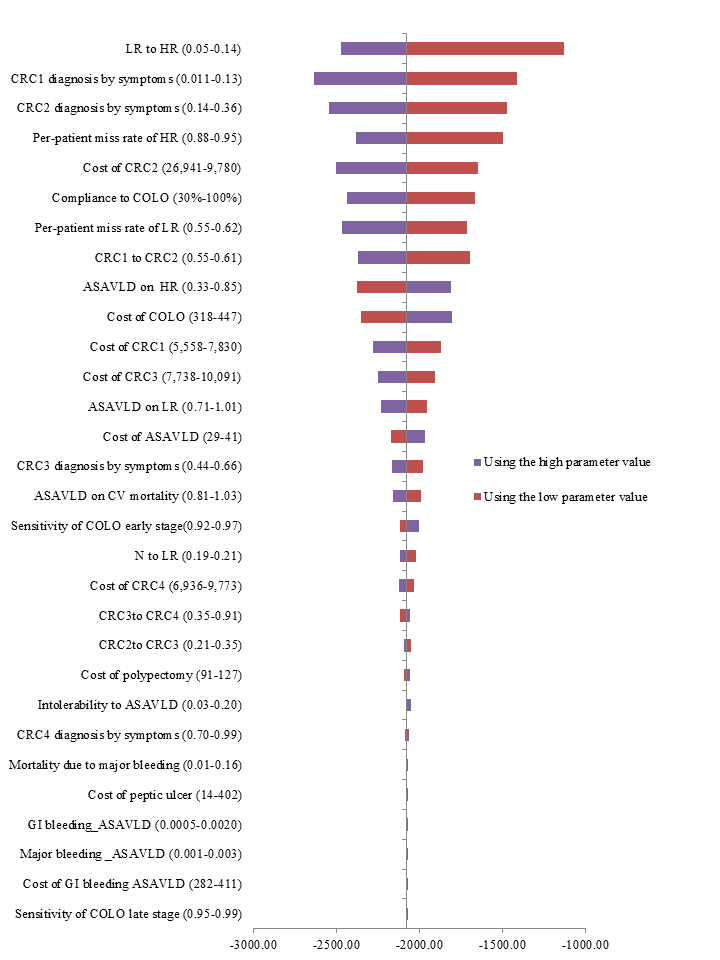


Abbreviations: ASAVLD: aspirin very-low-dose; COLO: colonoscopy, CRC: colorectal cancer;

GI: gastro intestinal; HR: high-risk adenoma state; LR: low-risk adenoma state; N: normal colon state

**S Figure 5.2 One-way sensitivity analysis for incremental quality-adjusted life years (QALYs)**


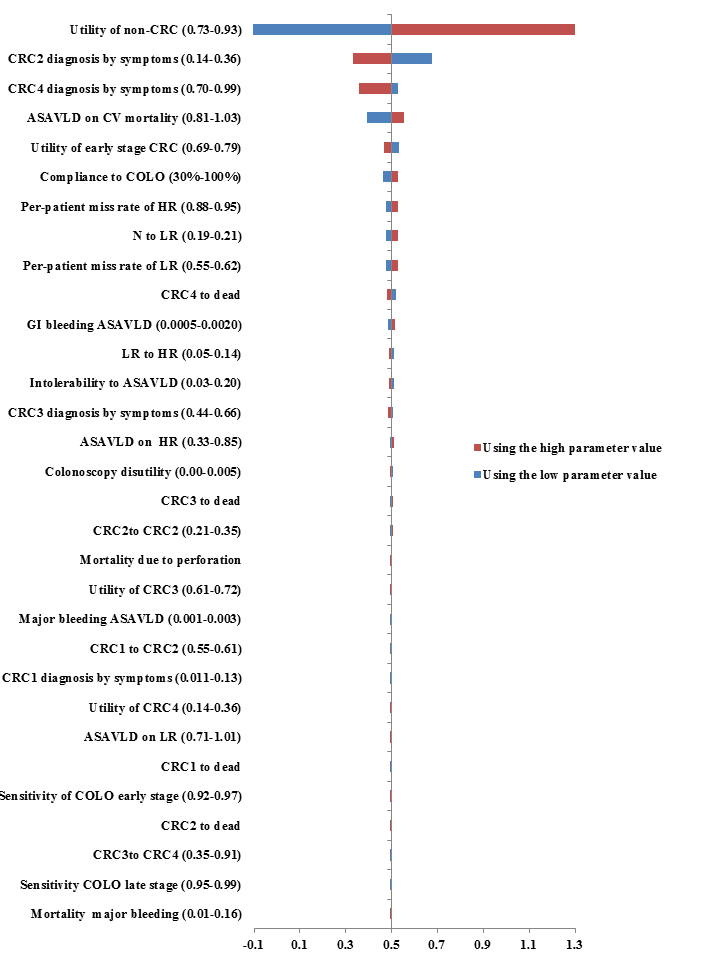


When the utility of 0.73 (lower limit) was used for non-CRC states, the combination strategy produced less gain in QALYs compared to no screening, without any changes in the incremental cost and LYs.

**S Figure 5.3 Cost-effectiveness plane of colorectal cancer preventive strategies compared to no screening**

**
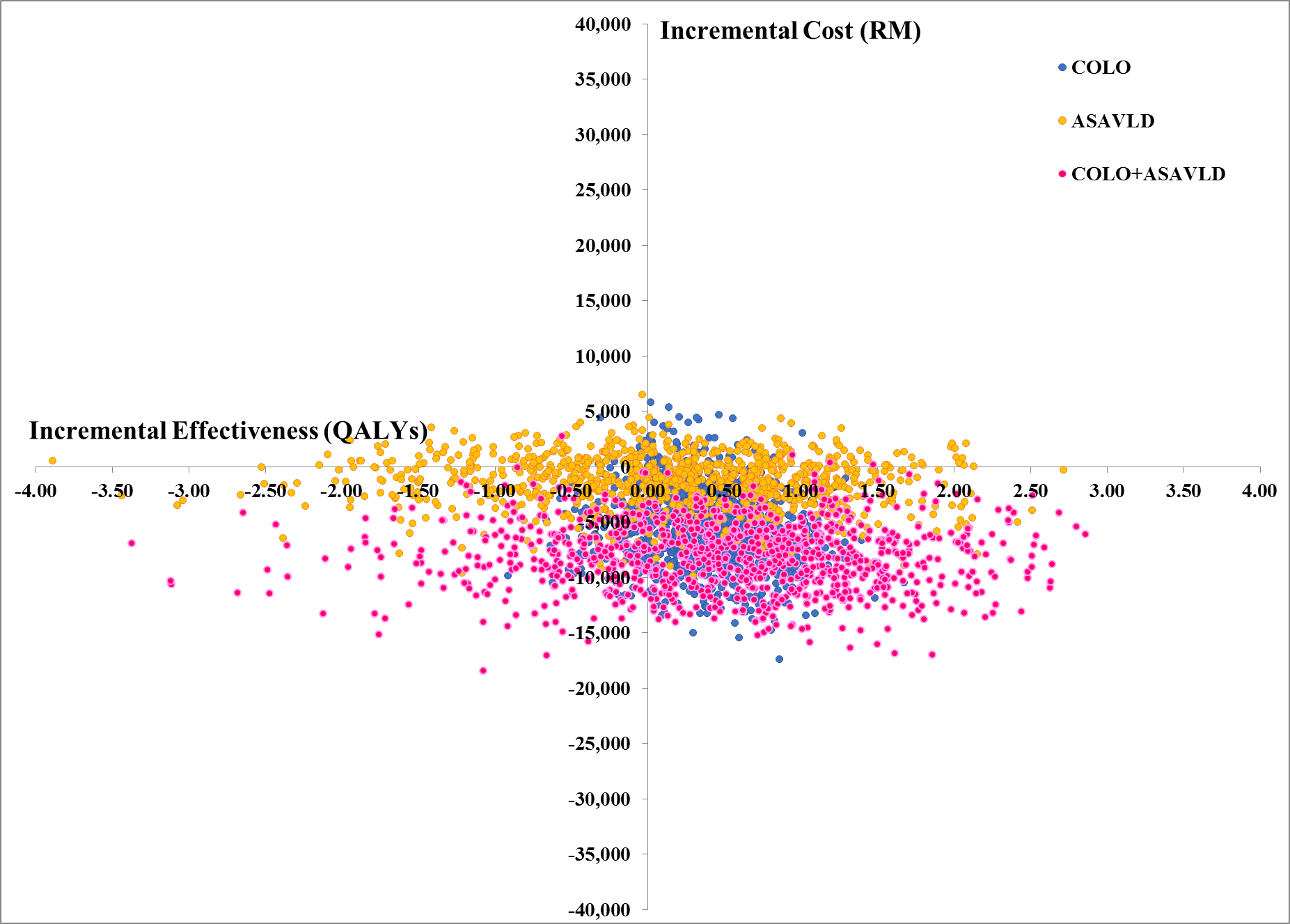
**

Abbreviations: ASAVLD, aspirin very-low-dose; COLO, colonoscopy; RM, Malaysian Ringgit

**S Table 5.2 Occurrence of CRC cases over lifetime for extending colonoscopy surveillance intervals**

| **Year** | **No screening** | **COLO every 3 years** | **ASAVLD+ COLO every 3 years** | **ASAVLD+ COLO every 5 years** | **ASAVLD+ COLO every 10 years** |
| --- | --- | --- | --- | --- | --- |
| 0 | 0 | 0 | 0 | 0 | 0 |
| 1 | 0 | 0 | 0 | 0 | 0 |
| 2 | 0 | 0 | 0 | 0 | 0 |
| 3 | 0 | 0 | 0 | 0 | 0 |
| 4 | 0 | 0 | 0 | 0 | 0 |
| 5 | 5 | 2 | 1 | 1 | 0 |
| 6 | 17 | 9 | 3 | 4 | 0 |
| 7 | 34 | 186 | 53 | 8 | 3 |
| 8 | 55 | 17 | 6 | 15 | 10 |
| 9 | 78 | 28 | 8 | 22 | 20 |
| 10 | 102 | 367 | 101 | 31 | 33 |
| 11 | 124 | 26 | 8 | 39 | 47 |
| 12 | 158 | 41 | 11 | 50 | 63 |
| 13 | 186 | 494 | 135 | 61 | 682 |
| 14 | 207 | 33 | 10 | 70 | 45 |
| 15 | 225 | 45 | 13 | 78 | 53 |
| 16 | 236 | 501 | 140 | 84 | 58 |
| 17 | 279 | 38 | 12 | 95 | 63 |
| 18 | 303 | 54 | 15 | 105 | 70 |
| 19 | 316 | 607 | 174 | 111 | 91 |
| 20 | 320 | 39 | 12 | 114 | 109 |
| 21 | 314 | 51 | 15 | 114 | 125 |
| 22 | 317 | 582 | 170 | 115 | 140 |
| 23 | 312 | 37 | 12 | 114 | 1306 |
| 24 | 302 | 49 | 15 | 110 | 74 |
| 25 | 289 | 530 | 158 | 105 | 77 |
| 26 | 269 | 33 | 11 | 98 | 78 |
| 27 | 261 | 44 | 14 | 94 | 80 |
| 28 | 247 | 56 | 17 | 89 | 81 |
| 29 | 230 | 69 | 23 | 80 | 89 |
| 30 | 212 | 82 | 31 | 71 | 96 |
| 31 | 186 | 90 | 39 | 63 | 102 |
| 32 | 164 | 97 | 47 | 56 | 107 |
| 33 | 144 | 101 | 55 | 49 | 918 |
| 34 | 125 | 102 | 61 | 37 | 66 |
| 35 | 109 | 101 | 65 | 28 | 54 |
| 36 | 82 | 86 | 59 | 21 | 42 |
| 37 | 62 | 72 | 53 | 16 | 40 |
| 38 | 47 | 60 | 47 | 12 | 34 |
| 39 | 36 | 49 | 41 | 9 | 29 |
| 40 | 27 | 40 | 36 | 7 | 26 |
| 41 | 21 | 32 | 31 | 5 | 23 |
| 42 | 16 | 26 | 26 | 4 | 21 |
| 43 | 12 | 21 | 22 | 3 | 18 |
| 44 | 9 | 16 | 18 | 2 | 16 |
| 45 | 7 | 13 | 15 | 2 | 14 |
| 46 | 5 | 10 | 12 | 1 | 12 |
| 47 | 4 | 8 | 10 | 1 | 10 |
| 48 | 3 | 6 | 8 | 1 | 9 |
| 49 | 2 | 5 | 7 | 0 | 7 |
| 50 | 2 | 4 | 5 | 0 | 6 |
| 51 | 1 | 2 | 3 | 0 | 5 |
| 52 | 0 | 1 | 1 | 0 | 4 |
| 53 | 0 | 0 | 1 | 0 | 2 |
| 54 | 0 | 0 | 0 | 0 | 1 |
| 55 | 0 | 0 | 0 | 0 | 1 |
| 56 | 0 | 0 | 0 | 0 | 0 |
| 57 | 0 | 0 | 0 | 0 | 0 |
| 58 | 0 | 0 | 0 | 0 | 0 |
| 59 | 0 | 0 | 0 | 0 | 0 |
| 60 | 0 | 0 | 0 | 0 | 0 |
|  | 6462 | 4962 | 1832 | 2879 | 5063 |
| **New CRC cases prevented in comparison with Surveillance colonoscopy every 3 years** | | | **3130** | **2083** | **Excess 101 new CRC cases** |
| **CRC prevention rate (%) in comparison with Surveillance colonoscopy every 3 years** | | | **63%** | **42%** | **-** |
| **CRC prevention rate (%) in comparison with no screening** | | | **72%** | **55%** | **22%** |

**Reference**

1. Short MW, Layton MC, Teer BN, et al. Colorectal Cancer Screening and Surveillance. Am Fam Physician 2015;91:93-100.

2. Hicks KA, Mahaffey KW, Mehran R, et al. 2017 Cardiovascular and Stroke Endpoint Definitions for Clinical Trials. Circulation 2018;137:961-972.

3. Wolff WI. Colonoscopy: history and development. Am J Gastroenterol 1989;84:1017-1025.

4. Sun et al. Withdrawal of COX-2 selective inhibitors rofecoxib and valdecoxib: impact on NSAID and gastroprotective drug prescribing and utilization. Curr Med Res Opin 2007;23:1859-1866.

5. Chubak J, Whitlock EP, Williams SB, et al. Aspirin for the Prevention of Cancer Incidence and Mortality: Systematic Evidence Reviews for the U.S. Preventive Services Task Force. Ann Intern Med 2016;164:814-825.

6. Qin T, Du M, Du H, et al. Folic acid supplements and colorectal cancer risk: meta-analysis of randomized controlled trials. Sci Rep 2015;5:12044.

7. Passarelli MN, Barry EL, Rees JR, et al. Folic acid supplementation and risk of colorectal neoplasia during long-term follow-up of a randomized clinical trial. Am J Clin Nutr August 2019.

8. Ladenheim J, Garcia G, Titzer D, et al. Effect of sulindac on sporadic colonic polyps. Gastroenterology 1995;108:1083-1087.

9. Baron JA, Beach M, Mandel JS, et al. Calcium supplements and colorectal adenomas. Polyp Prevention Study Group. Ann N Y Acad Sci 1999;889:138-145.

10. Baron JA, Beach M, Mandel JS, et al. Calcium supplements for the prevention of colorectal adenomas. Calcium Polyp Prevention Study Group. N Engl J Med 1999;340:101-107.

11. Benamouzig R, Deyra J, Martin A, et al. Daily soluble aspirin and prevention of colorectal adenoma recurrence: one-year results of the APACC trial. Gastroenterology 2003;125:328-336.

12. Benamouzig R, Uzzan B, Deyra J, et al. Prevention by daily soluble aspirin of colorectal adenoma recurrence: 4-year results of the APACC randomised trial. Gut 2012;61:255-261.

13. Baron JA, Sandler RS, Bresalier RS, et al. A randomized trial of rofecoxib for the chemoprevention of colorectal adenomas. Gastroenterology 2006;131:1674-1682.

14. Jaszewski R, Misra S, Tobi M, et al. Folic acid supplementation inhibits recurrence of colorectal adenomas: a randomized chemoprevention trial. World J Gastroenterol WJG 2008;14:4492-4498.

15. Benamouzig R, Uzzan B, Deyra J, et al. Prevention by Aspirin of Colorectal Adenoma Recurrence: Some Advances and Latest Results of the APACC Trial. Curr Colorectal Cancer Rep 2010;7:33-41.

16. Li ZY, Gu JL, Zeng Z, Shi W (2011). Clinical study of aspirin in the prevention of recurrence of colorectal adenoma in the elderly. Chinese J Med Guide, 13, 89, 2011.

17. Zhao T-Y, Tu J, Wang Y, et al. The Efficacy of Aspirin in Preventing the Recurrence of Colorectal Adenoma: a Renewed Meta-Analysis of Randomized Trials. Asian Pac J Cancer Prev APJCP 2016;17:2711-2717.

18. Takayama T, Nagashima H, Maeda M, et al. Randomized double-blind trial of sulindac and etodolac to eradicate aberrant crypt foci and to prevent sporadic colorectal polyps. Clin Cancer Res Off J Am Assoc Cancer Res 2011;17:3803-3811.

19. Bonelli L, Puntoni M, Gatteschi B, et al. Antioxidant supplement and long-term reduction of recurrent adenomas of the large bowel. A double-blind randomized trial. J Gastroenterol 2013;48:698-705.

20. McKeown-Eyssen G, Holloway C, Jazmaji V, et al. A randomized trial of vitamins C and E in the prevention of recurrence of colorectal polyps. Cancer Res 1988;48:4701-4705.

21. Roncucci L, Di Donato P, Carati L, et al. Antioxidant vitamins or lactulose for the prevention of the recurrence of colorectal adenomas. Colorectal Cancer Study Group of the University of Modena and the Health Care District 16. Dis Colon Rectum 1993;36:227-234.

22. Greenberg ER, Baron JA, Tosteson TD, et al. A Clinical Trial of Antioxidant Vitamins to Prevent Colorectal Adenoma. N Engl J Med 1994;331:141-147.

23. MacLennan R, Macrae F, Bain C, et al. Randomized trial of intake of fat, fiber, and beta carotene to prevent colorectal adenomas. J Natl Cancer Inst 1995;87:1760-1766.

24. Ponz de Leon M, Roncucci L. Chemoprevention of colorectal tumors: role of lactulose and of other agents. Scand J Gastroenterol Suppl 1997;222:72-75.

25. Hofstad B, Almendingen K, Vatn M, et al. Growth and recurrence of colorectal polyps: a double-blind 3-year intervention with calcium and antioxidants. Digestion 1998;59:148-156.

26. Wu K, Platz EA, Willett WC, et al. A randomized trial on folic acid supplementation and risk of recurrent colorectal adenoma. Am J Clin Nutr 2009;90:1623-1631.

27. Cole BF, Baron JA, Sandler RS, et al. Folic acid for the prevention of colorectal adenomas: A randomized clinical trial. JAMA 2007;297:2351-2359.

28. Baron JA, Cole BF, Sandler RS, et al. A Randomized Trial of Aspirin to Prevent Colorectal Adenomas. N Engl J Med 2003;348:891-899.

29. Meyskens FL, McLaren CE, Pelot D, et al. Difluoromethylornithine plus sulindac for the prevention of sporadic colorectal adenomas: a randomized placebo-controlled, double-blind trial. Cancer Prev Res Phila Pa 2008;1:32-38.

30. McAlister FA, Straus SE, Sackett DL, et al. Analysis and reporting of factorial trials: a systematic review. JAMA 2003;289:2545-2553.

31. JPT Higgins et al. A revised tool for assessing risk of bias in randomized trials. Cochrane Database of Systematic Reviews 2016;10(Suppl 1).

32. Higgins JPT, Green S. Cochrane Handbook for Systematic Reviews of Interventions Version 5.1.0 [Updated March 2011].The Cochrane Collaboration., 2011. http://handbook.cochrane.org/.

33. Higgins JPT, Thompson SG, Deeks JJ, et al. Measuring inconsistency in meta-analyses. BMJ 2003;327:557-560.

34. Hoaglin DC, Hawkins N, Jansen JP, et al. Conducting indirect-treatment-comparison and network-meta-analysis studies: report of the ISPOR Task Force on Indirect Treatment Comparisons Good Research Practices: part 2. Value Health J Int Soc Pharmacoeconomics Outcomes Res 2011;14:429-437.

35. Caldwell DM, Ades AE, Higgins JPT. Simultaneous comparison of multiple treatments: combining direct and indirect evidence. BMJ 2005;331:897-900.

36. Dias S, Welton NJ, Caldwell DM, et al. Checking consistency in mixed treatment comparison meta-analysis. Stat Med 2010;29:932-944.

37. Higgins J, Jackson D, Barrett J, et al. Consistency and inconsistency in network meta-analysis: concepts and models for multi-arm studies. Res Synth Methods 2012;3:98-110.

38. Chaimani A, Higgins JPT, Mavridis D, et al. Graphical Tools for Network Meta-Analysis in STATA. PLOS ONE 2013;8:e76654.

39. Puhan MA, Schünemann HJ, Murad MH, et al. A GRADE Working Group approach for rating the quality of treatment effect estimates from network meta-analysis. BMJ 2014;349:g5630.

40. Brok J, Thorlund K, Wetterslev J, et al. Apparently conclusive meta-analyses may be inconclusive—Trial sequential analysis adjustment of random error risk due to repetitive testing of accumulating data in apparently conclusive neonatal meta-analyses. Int J Epidemiol 2009;38:287-298.

41. Wetterslev J, Thorlund K, Brok J, et al. Trial sequential analysis may establish when firm evidence is reached in cumulative meta-analysis. J Clin Epidemiol 2008;61:64-75.

42. Thorlund K, Engstrøm J, Wetterslev J, Brok J, Imberger G, Gluud C. User Manual for Trial Sequential Analysis (TSA). Copenhagen Trial Unit, Centre for Clinical Intervention Research, Copenhagen, Denmark. 2011. p. 1-115. from www.ctu.dk/tsa.

43. Dulai PS, Singh S, Marquez E, et al. Chemoprevention of colorectal cancer in individuals with previous colorectal neoplasia: systematic review and network meta-analysis. BMJ 2016;355:i6188.

44. Martínez ME, Baron JA, Lieberman DA, et al. A pooled analysis of advanced colorectal neoplasia diagnoses after colonoscopic polypectomy. Gastroenterology 2009;136:832-841.

45. Nyaga VN, Arbyn M, Aerts M. Metaprop: a Stata command to perform meta-analysis of binomial data. Arch Public Health 2014;72:39.

46. L BJ, A AE, P A-C, et al. Grading quality of evidence and strength of recommendations in clinical practice guidelines. Allergy 2009;64:669-677.

47. Tappenden P, Chilcott J, Eggington S, et al. Option appraisal of population-based colorectal cancer screening programmes in England. Gut 2007;56:677-684.

48. Heitman SJ, Ronksley PE, Hilsden RJ, et al. Prevalence of adenomas and colorectal cancer in average risk individuals: a systematic review and meta-analysis. Clin Gastroenterol Hepatol Off Clin Pract J Am Gastroenterol Assoc 2009;7:1272-1278.

49. Sasieni PD, Shelton J, Ormiston-Smith N, et al. What is the lifetime risk of developing cancer?: the effect of adjusting for multiple primaries. Br J Cancer 2011;105:460-465.

50. Veettil SK, Lim KG, Chaiyakunapruk N, et al. Colorectal cancer in Malaysia: Its burden and implications for a multiethnic country. Asian J Surg 2016;0.

51. Clinical practice guidelines on Primary & Secondary Prevention of Cardiovascular Disease. Ministry of Health, Malaysia. 2017. http://www.acadmed.org.my. Accessed June 3, 2019.

52. Cooper K, Squires H, Carroll C, et al. Chemoprevention of colorectal cancer: systematic review and economic evaluation. Health Technol Assess Winch Engl 2010;14:1-206.

53. Lieberman DA, Rex DK, Winawer SJ, et al. Guidelines for colonoscopy surveillance after screening and polypectomy: a consensus update by the US Multi-Society Task Force on Colorectal Cancer. Gastroenterology 2012;143:844-857.

54. Clinical Practice Guidelines, Management of Colorectal Carcinoma, Ministry of Health, Malaysia. 2017. http://www.acadmed.org.my/index.cfm?menuid=67.

55. Jin J. Screening for Colorectal Cancer. JAMA 2016;315:2635-2635.

56. Global Health Observatory Data Repository, WHO, Apps.Who.Int. (2016). http://apps.who.int/gho/data/view.main.60990?lang=en) Global Health Observatory data repository, WHO, 2016.

57. Veettil SK, Nathisuwan S, Ching SM, et al. Efficacy and safety of celecoxib on the incidence of recurrent colorectal adenomas: a systematic review and meta-analysis. Cancer Manag Res 2019;11:561-571.

58. Dulai PS, Singh S, Marquez E, et al. Chemoprevention of colorectal cancer in individuals with previous colorectal neoplasia: systematic review and network meta-analysis. BMJ 2016;355:i6188.

59. Veettil SK, Lim KG, Ching SM, et al. Effects of aspirin and non-aspirin nonsteroidal anti-inflammatory drugs on the incidence of recurrent colorectal adenomas: a systematic review with meta-analysis and trial sequential analysis of randomized clinical trials. BMC Cancer 2017;17:763.

60. Veettil SK, Ching SM, Lim KG, et al. Effects of calcium on the incidence of recurrent colorectal adenomas: A systematic review with meta-analysis and trial sequential analysis of randomized controlled trials. Medicine (Baltimore) 2017;96:e7661.

61. Edge SB, Compton CC. The American Joint Committee on Cancer: the 7th Edition of the AJCC Cancer Staging Manual and the Future of TNM. Ann Surg Oncol 2010;17:1471-1474.

62. Aronsson M, Carlsson P, Levin L-Å, et al. Cost-effectiveness of high-sensitivity faecal immunochemical test and colonoscopy screening for colorectal cancer. Br J Surg 2017;104:1078-1086.

63. Naglie G, Krahn MD, Naimark D, et al. Primer on medical decision analysis: Part 3--Estimating probabilities and utilities. Med Decis Mak Int J Soc Med Decis Mak 1997;17:136-141.

64. Miller DK, Homan SM. Determining transition probabilities: confusion and suggestions. Med Decis Mak Int J Soc Med Decis Mak 1994;14:52-58.

65. Brenner H, Altenhofen L, Stock C, et al. Natural history of colorectal adenomas: birth cohort analysis among 3.6 million participants of screening colonoscopy. Cancer Epidemiol Biomarkers Prev 2013;22:1043-1051.

66. Heresbach D, Barrioz T, Lapalus MG, et al. Miss rate for colorectal neoplastic polyps: a prospective multicenter study of back-to-back video colonoscopies. Endoscopy 2008;40:284-290.

67. Aronchick CA, Lipshutz WH, Wright SH, et al. A novel tableted purgative for colonoscopic preparation: efficacy and safety comparisons with Colyte and Fleet Phospho-Soda. Gastrointest Endosc 2000;52:346-352.

68. Kim NH, Jung YS, Jeong WS, et al. Miss rate of colorectal neoplastic polyps and risk factors for missed polyps in consecutive colonoscopies. Intest Res 2017;15:411-418.

69. Shin JG, Kim HW, Park SB, et al. Polyp missing rate and its associated risk factors of referring hospitals for endoscopic resection of advanced colorectal neoplasia. Medicine (Baltimore) 2017;96.

70. Chang JY, Moon CM, Lee HJ, et al. Predictive factors for missed adenoma on repeat colonoscopy in patients with suboptimal bowel preparation on initial colonoscopy: A KASID multicenter study. PLOS ONE 2018;13:e0195709.

71. Xiang L. Risk factors associated with missed colorectal flat adenoma: A multicenter retrospective tandem colonoscopy study. World J Gastroenterol 2014;20:10927.

72. Hong SN, Sung IK, Kim JH, et al. The Effect of the Bowel Preparation Status on the Risk of Missing Polyp and Adenoma during Screening Colonoscopy: A Tandem Colonoscopic Study. Clin Endosc 2012;45:404-411.

73. Pickhardt PJ, Hassan C, Halligan S, et al. Colorectal cancer: CT colonography and colonoscopy for detection--systematic review and meta-analysis. Radiology 2011;259:393-405.

74. Ishikawa H, Mutoh M, Suzuki S, et al. The preventive effects of low-dose enteric-coated aspirin tablets on the development of colorectal tumours in Asian patients: a randomised trial. Gut 2014;63:1755-1759.

75. Mahmoud AN, Gad MM, Elgendy AY, et al. Efficacy and safety of aspirin for primary prevention of cardiovascular events: a meta-analysis and trial sequential analysis of randomized controlled trials. Eur Heart J 2019;40:607-617.

76. National Collaborating Centre for Chronic Conditions. Osteoarthritis: National clinical guideline for care and management in adults. London: Royal College of Physicians; 2008.

77. Veettil SK, Jinatongthai P, Nathisuwan S, et al. E fficacy and safety of chemo-preventive agents on colorectal cancer incidence and mortality: systematic review and network meta-analysis. Clin Epidemiol 2018;10:1433-1445.

78. Whitlock EP, Williams SB, Burda BU, et al. Aspirin Use in Adults: Cancer, All-Cause Mortality, and Harms: A Systematic Evidence Review for the U.S. Preventive Services Task Force. Rockville (MD): Agency for Healthcare Research and Quality (US), 2015. http://www.ncbi.nlm.nih.gov/books/NBK321643/. Accessed December 14, 2016.

79. Dubé C, Rostom A, Lewin G, et al. The use of aspirin for primary prevention of colorectal cancer: a systematic review prepared for the U.S. Preventive Services Task Force. Ann Intern Med 2007;146:365-375.

80. Omar A, Ganapathy SS, Anuar MFM, et al. Cause-specific mortality estimates for Malaysia in 2013: results from a national sample verification study using medical record review and verbal autopsy. BMC Public Health 2019;19:110.

81. Kong C-K, Roslani AC, Law C-W, et al. Impact of socio-economic class on colorectal cancer patient outcomes in Kuala Lumpur and Kuching, Malaysia. Asian Pac J Cancer Prev APJCP 2010;11:969-974.

82. Magaji BA, Moy FM, Roslani AC, et al. Health-related quality of life among colorectal cancer patients in Malaysia: a study protocol. BMC Cancer 2012;12.

83. Hassan MRA, Suan MAM, Soelar SA, et al. Survival Analysis and Prognostic Factors for Colorectal Cancer Patients in Malaysia. Asian Pac J Cancer Prev APJCP 2016;17:3575-3581.

84. Ghazali AK, Musa KI, Naing NN, et al. Prognostic factors in patients with colorectal cancer at Hospital Universiti Sains Malaysia. Asian J Surg 2010;33:127-133.

85. Ness RM, Holmes AM, Klein R, et al. Utility valuations for outcome states of colorectal cancer. Am J Gastroenterol 1999;94:1650-1657.

86. Ramsey SD, Berry K, Moinpour C, et al. Quality of life in long term survivors of colorectal cancer. Am J Gastroenterol 2002;97:1228-1234.

87. NICE. Guide to the methods of technology apprasial. London: NICE; 2008.

88. Neumann PJ, Sanders GD, Russell LB, et al. Cost-Effectiveness in Health and Medicine. Oxford University Press, 2016.

89. Shafie AA, Hassali MA, Liau SY. A cross-sectional validation study of EQ-5D among the Malaysian adult population. Qual Life Res Int J Qual Life Asp Treat Care Rehabil 2011;20:593-600.

90. Siti Fatimah MN, Rosminah M, Suhaimi AW, et al. The measurement of quality of life among population within the crowd: a case study among malaysian pilgrims in makkah. Malaysian Journal of Public Health Medicine 2017;17 (1): 137-145.

91. Saini SD, Schoenfeld P, Vijan S. Surveillance colonoscopy is cost-effective for patients with adenomas who are at high risk of colorectal cancer. Gastroenterology 2010;138:2292-2299.

92. Private health care facilities and services (Private hospitals and other private healthcare facilities) (amendment) order. 2013. https://new.medicine.com.my/2014/03/the-amended-fee-schedule/.

93. The Consumer Price Guide (CPG), Pharmaceutical Services Program, Ministry of Health, Malaysia. https://www.pharmacy.gov.my/v2/en/apps/drug-price.

94. Azzani M, Dahlui M, Wan Ishak WZ, Roslani AC, Su TT. Provider costs of treating colorectal cancer in government hospital of Malaysia. Malays J Med Sci. 2019;26(1):73–86.

95. Mohd et al.,. Cost analysis of colorectal cancer (CRC) management in UKM Medical Centre using clinical pathway. BMC Public Health 2012;12:A40.

96. Ezat SW et al.,. Economic evaluation of monoclonal antibody in the management of colorectal cancer in Malaysia. BMC Health Serv Res 2012;12:P3.

97. Clinical Practice Guidelines, Management of Colorectal Carcinoma, Ministry of Health, Malaysia, 2017. http://www.acadmed.org.my/index.cfm?menuid=67.

98. Pok LSL, Shabaruddin FH, Dahlui M, et al. Clinical and economic implications of upper gastrointestinal adverse events in Asian rheumatological patients on long-term non-steroidal anti-inflammatory drugs. Int J Rheum Dis 2018;21:943-951.

99. Consumer Price Index Malaysia, Department of Statistics Malaysia. https://www.dosm.gov.my/v1/.

100. Bonithon-Kopp C, Kronborg O, Giacosa A, et al. Calcium and fibre supplementation in prevention of colorectal adenoma recurrence: a randomised intervention trial. European Cancer Prevention Organisation Study Group. Lancet Lond Engl 2000;356:1300-1306.

101. Sandler RS, Halabi S, Baron JA, et al. A Randomized Trial of Aspirin to Prevent Colorectal Adenomas in Patients with Previous Colorectal Cancer. N Engl J Med 2003;348:883-890.

102. Arber N, Eagle CJ, Spicak J, et al. Celecoxib for the Prevention of Colorectal Adenomatous Polyps. N Engl J Med 2006;355:885-895.

103. Bertagnolli MM, Hsu M, Hawk ET, et al. Statin use and colorectal adenoma risk: results from the adenoma prevention with celecoxib trial. Cancer Prev Res Phila Pa 2010;3:588-596.

104. Logan RFA, Grainge MJ, Shepherd VC, et al. Aspirin and folic acid for the prevention of recurrent colorectal adenomas. Gastroenterology 2008;134:29-38.

105. Chu D.Z.J., Hussey M.A., Alberts D.S., et al. Colorectal chemoprevention pilot study (SWOG-9041), randomized and placebo controlled: The importance of multiple luminal lesions. Clinical Colorectal Cancer. http://www.journals.elsevier.com/clinical-colorectal-cancer. Published 2011.

106. Baron JA, Barry EL, Mott LA, et al. A Trial of Calcium and Vitamin D for the Prevention of Colorectal Adenomas. J Med 2015;373:1519-1530.

107. Thompson PA, Ashbeck EL, Roe DJ, et al. Celecoxib for the Prevention of Colorectal Adenomas: Results of a Suspended Randomized Controlled Trial. JNCI J Natl Cancer Inst 2016;108.

108. Pommergaard H-C, Burcharth J, Rosenberg J, et al. Aspirin, Calcitriol, and Calcium Do Not Prevent Adenoma Recurrence in a Randomized Controlled Trial. Gastroenterology 2016;150:114-122.

109. Hull MA, Sprange K, Hepburn T, et al. Eicosapentaenoic acid and aspirin, alone and in combination, for the prevention of colorectal adenomas (seAFOod Polyp Prevention trial): a multicentre, randomised, double-blind, placebo-controlled, 2 × 2 factorial trial. Lancet Lond Engl 2018;392:2583-2594.

110. Riley RD, Higgins JPT, Deeks JJ. Interpretation of random effects meta-analyses. BMJ 2011;342:d549.
